# PqsE Expands and Differentially Modulates the RhlR Quorum Sensing Regulon in Pseudomonas aeruginosa

**DOI:** 10.1128/spectrum.00961-22

**Published:** 2022-05-23

**Authors:** Morgana Letizia, Marta Mellini, Alessandra Fortuna, Paolo Visca, Francesco Imperi, Livia Leoni, Giordano Rampioni

**Affiliations:** a Department of Science, Roma Tre University, Rome, Italy; b IRCCS Fondazione Santa Lucia, Rome, Italy; Emory University School of Medicine

**Keywords:** *Pseudomonas aeruginosa*, quorum sensing, virulence, pyocyanin, PqsE, RhlR, gene regulation, RNA-seq

## Abstract

In the opportunistic pathogen Pseudomonas aeruginosa, many virulence traits are finely regulated by quorum sensing (QS), an intercellular communication system that allows the cells of a population to coordinate gene expression in response to cell density. The key aspects underlying the functionality of the complex regulatory network governing QS in P. aeruginosa are still poorly understood, including the interplay between the effector protein PqsE and the transcriptional regulator RhlR in controlling the QS regulon. Different studies have focused on the characterization of PqsE- and RhlR-controlled genes in genetic backgrounds in which RhlR activity can be modulated by PqsE and *pqsE* expression is controlled by RhlR, thus hampering identification of the distinct regulons controlled by PqsE and RhlR. In this study, a P. aeruginosa PAO1 mutant strain with deletion of multiple QS elements and inducible expression of *pqsE* and/or *rhlR* was generated and validated. Transcriptomic analyses performed on this genetic background allowed us to unambiguously define the regulons controlled by PqsE and RhlR when produced alone or in combination. Transcriptomic data were validated via reverse transcription-quantitative PCR (RT-qPCR) and transcriptional fusions. Overall, our results showed that PqsE has a negligible effect on the P. aeruginosa transcriptome in the absence of RhlR, and that multiple RhlR subregulons exist with distinct dependency on PqsE. Overall, this study contributes to untangling the regulatory link between the *pqs* and *rhl* QS systems mediated by PqsE and RhlR and clarifying the impact of these QS elements on the P. aeruginosa transcriptome.

**IMPORTANCE** The ability of Pseudomonas aeruginosa to cause difficult-to-treat infections relies on its capacity to fine-tune the expression of multiple virulence traits via the *las*, *rhl*, and *pqs* QS systems. Both the *pqs* effector protein PqsE and the *rhl* transcriptional regulator RhlR are required for full production of key virulence factors *in vitro* and pathogenicity *in vivo*. While it is known that PqsE can stimulate the ability of RhlR to control some virulence factors, no data are available to allow clear discrimination of the PqsE and RhlR regulons. The data produced in this study demonstrate that PqsE mainly impacts the P. aeruginosa transcriptome via an RhlR-dependent pathway and splits the RhlR regulon into PqsE-dependent and PqsE-independent subregulons. Besides contributing to untangling of the complex QS network of P. aeruginosa, our data confirm that both PqsE and RhlR are suitable targets for the development of antivirulence drugs.

## INTRODUCTION

Quorum sensing (QS) is a cell-to-cell communication system based on the production, secretion, and perception of signal molecules. QS enables bacterial cells to behave as a community, coordinating gene expression and the display of related phenotypes at the population level depending on cell density and environmental cues. QS networks are widespread in bacteria, driving essential traits for pathogenicity such as the production of virulence factors, biofilm formation, group motility, and antibiotic resistance (1–3).

The Gram-negative human pathogen Pseudomonas aeruginosa is considered a model organism for QS and quorum-quenching studies. P. aeruginosa possesses a sophisticated QS network consisting of three main interacting systems which, overall, control over 10% of the P. aeruginosa genome. P. aeruginosa QS plays a crucial role in biofilm formation and the regulation of multiple virulence factors, including pyocyanin, rhamnolipids, hydrogen cyanide, LasB elastase, LasA protease, and LecA and LecB lectins (3, 4). Consequently, QS interference is considered a promising strategy for reducing P. aeruginosa pathogenicity (5–9).

P. aeruginosa has two *N*-acyl homoserine lactone (AHL)-dependent QS circuits, namely, the *las* and *rhl* systems, based on the LasR and RhlR transcriptional regulators activated by *N*-3-oxo-dodecanoyl-homoserine lactone (3OC_12_-HSL) and *N*-butanoyl-homoserine lactone (C_4_-HSL) signal molecules, respectively. The synthesis of 3OC_12_-HSL and C_4_-HSL is directed by the LasI and RhlI synthases, respectively. Once activated, LasR and RhlR regulate the transcription of multiple target genes (2). A third QS system, *pqs*, uses the 2-alkyl-4-quinolones (AQs) molecules 2-heptyl-3-hydroxy-4-quinolone (also known as the Pseudomonas quinolone signal, PQS) and 2-heptyl-4-hydroxyquinoline (HHQ) as QS signals. HHQ synthesis requires the enzymes encoded by the first four genes of the *pqsABCDE*-*phnAB* operon. The PqsH monooxygenase, encoded by the *pqsH* gene, converts HHQ to PQS. Both HHQ and PQS bind to and activate the transcriptional regulator PqsR (10–11). Unlike LasR and RhlR, activated PqsR does not act as a global regulator, but mainly promotes transcription of the *pqsABCDE-phnAB* operon by activating the P*pqsA* promoter (12). This autoregulatory loop results in the amplified synthesis of AQs and increased production of the effector protein PqsE, encoded by the fifth gene of the *pqsABCDE-phnAB* operon. Despite the fact that PqsE is required for full virulence in P. aeruginosa (13–15), its mechanism of action has not yet been clarified.

Structural studies revealed that PqsE has a typical metallo-β-lactamase fold, without obvious DNA-binding motifs (16). PqsE has thioesterase activity involved in the hydrolysis of 2-aminobenzoyl-acetyl-CoA (2-ABA-CoA) to 2-aminobenzoyl-acetate (2-ABA), an intermediate of HHQ and PQS synthesis (17). However, HHQ and PQS levels are unaltered in P. aeruginosa *pqsE*-deletion mutants relative to those in their isogenic wild-type strains, as other thioesterases can substitute for PqsE activity (14, 17, 18). Intriguingly, mutations in its catalytic site and inhibitors of its thioesterase activity do not inhibit the ability of PqsE to promote the expression of virulence genes, such as those involved in pyocyanin and rhamnolipid production, indicating that PqsE is a multifunctional protein (19–23). Different studies have shown that PqsE-dependent control of these virulence factors requires RhlR (12, 13, 24–27). Furthermore, deletion of either *pqsE* or *rhlR* in P. aeruginosa causes a strong attenuation of virulence in different plant and animal infection models (12–15, 28, 29).

Great effort has been directed towards uncovering the mechanism(s) by which PqsE and RhlR impact the regulation of common target genes. It has been demonstrated that the RhlR/C_4_-HSL complex can trigger gene transcription in the absence of PqsE (26, 30) and that PqsE stimulates the RhlR/C_4_-HSL-dependent activation of pyocyanin and rhamnolipid genes (15, 26, 31, 32). In the last few years, possible mechanisms have been proposed to explain this regulatory link, including the synthesis by PqsE of an alternative RhlR ligand, which would activate the expression of some target genes even in the absence of C_4_-HSL (15, 31), direct interaction between PqsE and RhlR, which would increase RhlR affinity to target promoters (22, 23), and a PqsE-mediated increase in RhlR intracellular abundance, which was proposed to occur independently of alterations in *rhlR* gene transcription and mRNA translation (33).

Transcriptomic analyses showed that PqsE is required for the expression of more than 100 genes independent of the other elements of the *pqs* QS system, many of which encode virulence factors (12–14, 23). However, since previous experiments were performed in RhlR-proficient genetic backgrounds, it is not clear whether and to what extent PqsE can control gene expression independently of RhlR. Moreover, the impact of RhlR on the P. aeruginosa transcriptome has never been investigated in a *pqsE-*negative background; hence, the genes regulated by RhlR in a PqsE-dependent or PqsE-independent manner have not yet been defined.

Outlining the specific effects of PqsE and RhlR on the P. aeruginosa transcriptome is a challenging task since the *las*, *rhl*, and *pqs* QS systems are closely interconnected (3, 34). Indeed, the LasR/3OC_12_-HSL complex exerts a positive control on the expression of the *rhlR*, *rhlI*, *pqsR*, and *pqsH* genes, thus stimulating activation of both the *rhl* and *pqs* QS systems (18, 35–39). The *rhl* system, in turn, has positive and negative effects on *lasI* and *pqs* gene expression, respectively (38–42). Finally, the *pqs* QS system has a positive effect on the expression of *rhlR* and *rhlI* (24, 43).

In this context, to fill the gap of knowledge regarding the specific contributions of PqsE and RhlR to the P. aeruginosa transcriptome, in this study we generated a P. aeruginosa PAO1 mutant strain with deletions in all the genes involved in the synthesis and reception of the QS signal molecules 3OC_12_-HSL, C_4_-HSL, and HHQ/PQS, which carries chromosomally integrated and episomal genetic elements for inducible expression of *pqsE* and/or *rhlR*, respectively. RNA-seq analysis performed in this genetic background allowed us to untangle the roles played by PqsE and RhlR on the P. aeruginosa transcriptome and classify the RhlR-controlled genes into distinct subregulons based on their PqsE dependency.

## RESULTS

### Generation of suitable genetic backgrounds to investigate the PqsE, RhlR, and PqsE-RhlR regulons.

**FIG 1:**
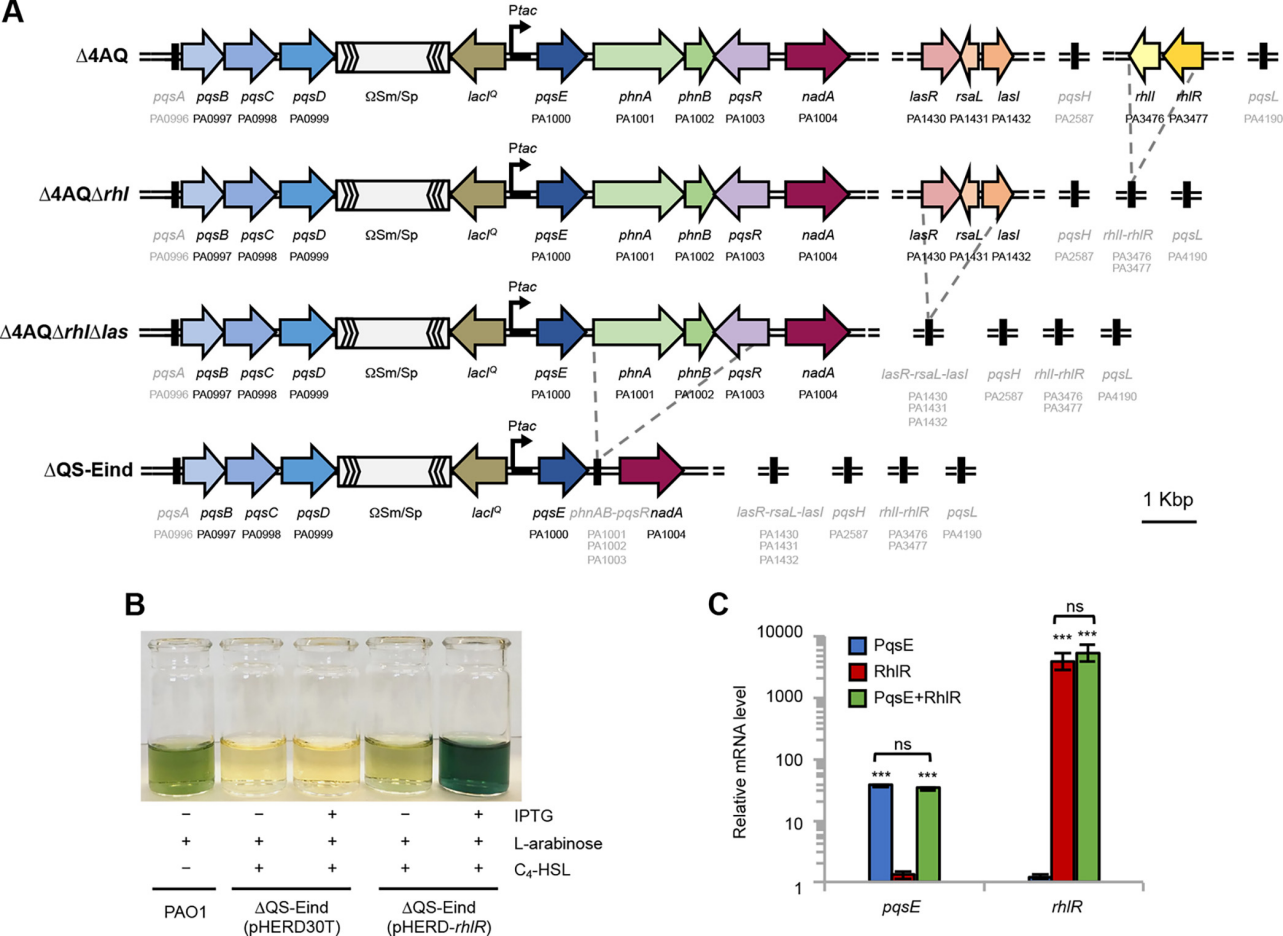
Genetic organization and validation of the quorum-sensing (QS)-defective strains generated in this study. (A) Schematic representation of the QS gene *loci* in the Δ4AQ strain (12) and in the derivative mutants Δ4AQΔ*rhl*, Δ4AQΔ*rhlΔlas*, and *Δ*QS-Eind. The PA number is indicated below the genes according to the Pseudomonas Genome Database (102). Black thick solid lines indicate gene deletions; names and PA numbers of deleted genes are shown in light gray; ΩSm/Sp, Ω45 gene cassette containing a streptomycin/spectinomycin resistance gene. (B) Image of cell-free supernatants from cultures of the wild-type P. aeruginosa PAO1 strain (PAO1) and its isogenic ΔQS-Eind(pHERD30T) and ΔQS-Eind(pHERD-*rhlR*) mutants grown in LB supplemented (+) or not (–) with 500 μM isopropyl-β-D-1-thiogalactopyranoside (IPTG), 0.1% (wt/vol) l-arabinose, and/or 10 μM N-butanoyl-homoserine lactone (C_4_-HSL). A representative picture from three independent experiments is shown. (C) Histogram reporting the relative levels of *pqsE* and *rhlR* mRNA measured by reverse transcription-quantitative PCR (RT-qPCR) in the ΔQS-Eind(pHERD30T) strain grown in LB supplemented with 0.1% (wt/vol) L-arabinose, 10 μM C_4_-HSL, and 500 μM IPTG (*pqsE*-expressing condition, PqsE, blue bars), ΔQS-Eind(pHERD-*rhlR*) strain grown in LB supplemented with 0.1% (wt/vol) L-arabinose and 10 μM C_4_-HSL (*rhlR*-expressing condition, RhlR, red bars), and ΔQS-Eind(pHERD-*rhlR*) strain grown in LB supplemented with 0.1% (wt/vol) L-arabinose, 10 μM C_4_-HSL, and 500 μM IPTG (*pqsE*-*rhlR*-expressing condition, PqsE + RhlR, green bars), relative to that in the ΔQS-Eind(pHERD30T) strain grown in LB supplemented with 0.1% (wt/vol) L-arabinose and 10 μM C_4_-HSL (baseline condition). The average of three independent experiments is reported with standard deviation (SD). Asterisks indicate statistically significant differences (*P* < 0.001) with respect to the baseline condition. Differences in *pqsE* levels between the *pqsE*- and *pqsE*-*rhlR*-expressing conditions, and in *rhlR* levels between the *rhlR*- and *pqsE*-*rhlR*-expressing conditions, are not statistically significant (ns).

To investigate the impacts of PqsE and/or RhlR on the transcriptome of P. aeruginosa PAO1 (Nottingham collection), we generated QS-defective mutants in which the expression of *pqsE* and/or *rhlR* could be induced by isopropyl-β-d-1-thiogalactopyranoside (IPTG) and/or l-arabinose, respectively. Briefly, the P. aeruginosa PAO1 mutant strain Δ4AQ (12), which carries in-frame deletions of the *pqsA*, *pqsH*, and *pqsL* genes, and a chromosomally integrated construct for IPTG-inducible expression of *pqsE* (Fig. 1A), had three gene *loci* sequentially deleted, including (i) *rhlI* and *rhlR*, (ii) *lasI*, *rsaL*, and *lasR*, and (iii) *phnA*, *phnB*, and *pqsR*. As expected, the resulting mutant strain, named ΔQS-Eind (Fig. 1A), was unable to produce QS signal molecules or the QS-controlled virulence factor pyocyanin (Fig. S1 in the supplemental material). To express *rhlR* in the ΔQS-Eind strain, the *rhlR* coding region was cloned under the control of the l-arabinose-controlled P_BAD_ promoter in the pHERD30T vector (44). The functionality of the resulting pHERD-*rhlR* plasmid was verified by assessing the ability of l-arabinose to restore wild-type levels of C_4_-HSL and pyocyanin production in a P. aeruginosa PAO1 Δ*rhlR* mutant (14) (Fig. S2). Next, pHERD30T and pHERD-*rhlR* were independently introduced into the ΔQS-Eind mutant strain.

Preliminary analyses confirmed that high pyocyanin production in ΔQS-Eind(pHERD-*rhlR*) requires concomitant, IPTG-dependent expression of *pqsE* and l-arabinose-dependent expression of *rhlR* in the presence of exogenous C_4_-HSL (Fig. 1B), in agreement with literature data (15, 26, 29). Some pyocyanin production was also observed upon *rhlR* expression in the absence of *pqsE*, in accordance with a previous report (32). In addition, reverse transcription-quantitative PCR (RT-qPCR) analyses confirmed that *pqsE* transcription was induced only in the ΔQS-Eind(pHERD30T) and ΔQS-Eind(pHERD-*rhlR*) strains grown in the presence of IPTG, whereas transcription of *rhlR* was promoted by l-arabinose only in the ΔQS-Eind(pHERD-*rhlR*) strain. Importantly, under the tested conditions, IPTG-dependent expression of *pqsE* did not affect l-arabinose-dependent expression of *rhlR*, and *vice versa* (Fig. 1C).

Overall, these analyses confirmed that the ΔQS-Eind(pHERD30T) and ΔQS-Eind(pHERD-*rhlR*) strains are suitable genetic backgrounds for investigating the impacts of PqsE and RhlR on the P. aeruginosa transcriptome when produced alone or in combination.

### RNA-seq analyses to define the regulons controlled by PqsE and RhlR when produced alone or in combination.

The specific contributions of PqsE and RhlR, alone or in combination, on the P. aeruginosa transcriptome were determined by RNA-seq analysis of the following cultures, all grown in LB supplemented with 0.1% (wt/vol) l-arabinose and 10 μM C_4_-HSL: (i) ΔQS-Eind(pHERD30T), in which *pqsE* and *rhlR* were not expressed (baseline); (ii) ΔQS-Eind(pHERD30T) with 500 μM IPTG, in which only *pqsE* was expressed (PqsE); (iii) ΔQS-Eind(pHERD-*rhlR*), in which only *rhlR* was expressed (RhlR); and (iv) ΔQS-Eind(pHERD-*rhlR*) with IPTG, in which both *pqsE* and *rhlR* were expressed (PqsE + RhlR).

Alteration of gene expression levels caused by the addition of IPTG to the ΔQS-Eind(pHERD30T) strain should allow the identification of genes specifically regulated by PqsE, as IPTG *per se* does not affect the P. aeruginosa PAO1 transcriptome (14). Conversely, while a general effect of l-arabinose on gene expression cannot be excluded, the transcriptional profiles of the ΔQS-Eind(pHERD30T) and ΔQS-Eind(pHERD-*rhlR*) strains, both grown in the presence of l-arabinose, have been compared to unequivocally identify RhlR-regulated genes. Furthermore, since AHL signal molecules can alter gene expression in P. aeruginosa regardless of their cognate QS receptors (45), synthetic C_4_-HSL was added to all the tested cultures to activate RhlR (when present) and avoid including the RhlR regulon genes whose expression could be altered in response to C_4_-HSL independently of RhlR.

Following statistical validation of the data set, only genes with a fold change (FC) of ≥ ±2.0 and an adjusted *P* value of <0.05 were considered for further analysis (46). Briefly, 4, 201, and 393 genes were identified as differentially regulated in the ΔQS-Eind mutant upon expression of *pqsE* alone, *rhlR* alone, or both *pqsE* and *rhlR*, respectively. The full list of genes controlled by PqsE and/or RhlR is reported in Table S1 in the supplemental material. These results will be detailed and furthered in the following sections.

### (i) PqsE has a limited effect on the P. aeruginosa transcriptome in the absence of RhlR.

**FIG 2:**
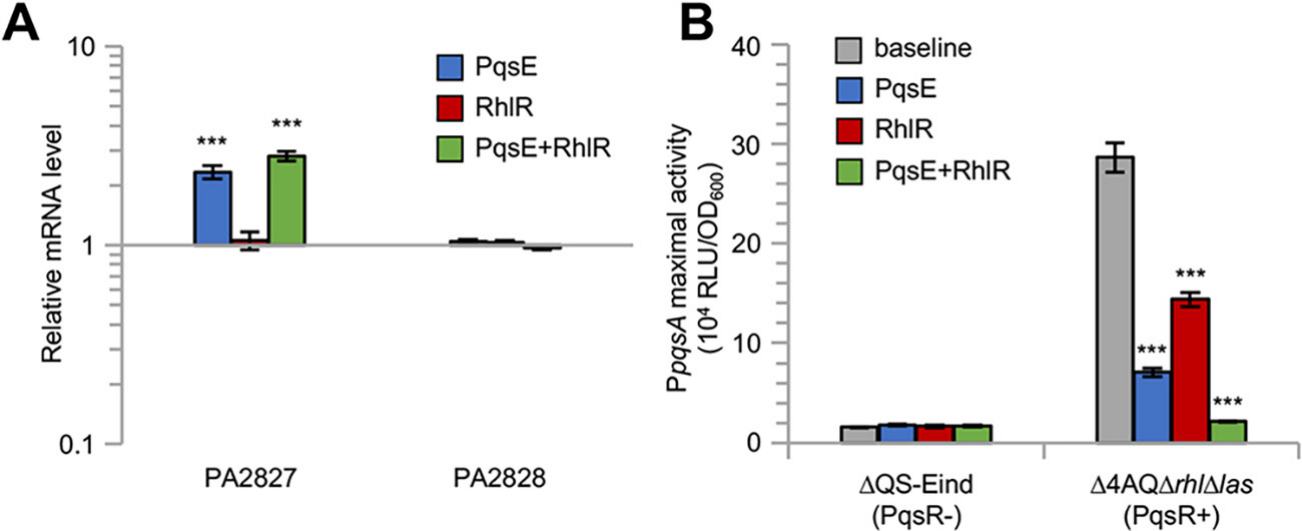
Genes controlled by PqsE in the absence of RhlR. (A) Histogram reporting the relative levels of (RT-qPCR) PA2827 and PA2828 mRNA measured by RT-qPCR in the ΔQS-Eind(pHERD30T) strain grown in LB supplemented with 0.1% (wt/vol) L-arabinose, 10 μM C_4_-HSL, and 500 μM IPTG (*pqsE*-expressing condition, PqsE, blue bars), ΔQS-Eind(pHERD-*rhlR*) strain grown in LB supplemented with 0.1% (wt/vol) L-arabinose and 10 μM C_4_-HSL (*rhlR*-expressing condition, RhlR, red bars), and ΔQS-Eind(pHERD-*rhlR*) strain grown in LB supplemented with 0.1% (wt/vol) L-arabinose, 10 μM C_4_-HSL, and 500 μM IPTG (*pqsE*-*rhlR*-expressing condition, PqsE + RhlR, green bars), relative to that in the ΔQS-Eind(pHERD30T) strain grown in LB supplemented with 0.1% (wt/vol) L-arabinose and 10 μM C_4_-HSL (baseline condition). The average of three independent experiments is reported with SD. Asterisks indicate statistically significant differences (*P* < 0.001) with respect to the baseline condition. (B) Histogram reporting the maximum P*pqsA*::*lux* activity measured in the ΔQS-Eind and Δ4AQΔ*rhlΔlas* strains carrying the pHERD30T empty vector and grown in LB supplemented with 0.1% (wt/vol) L-arabinose and 10 μM C_4_-HSL, in the absence (baseline condition, gray bars) or presence of 500 μM IPTG (*pqsE*-expressing condition, PqsE, blue bars), or carrying the pHERD-*rhlR* plasmid and grown in LB supplemented with 0.1% (wt/vol) L-arabinose and 10 μM C_4_-HSL, in the absence (*rhlR*-expressing condition, RhlR, red bars) or presence of 500 μM IPTG (*pqsE*-*rhlR*-expressing condition, PqsE+RhlR, green bars). RLU, relative light units. The average of three independent experiments is reported with SD. Asterisks indicate statistically significant differences (*P* < 0.001) with respect to the Δ4AQΔ*rhlΔlas* baseline condition.

The RNA-seq experiment revealed that the mRNA levels of only 4 genes were altered upon *pqsE* expression in the ΔQS-Eind(pHERD30T) strain, namely, *pqsE*, *nadA*, PA2827, and PA2828 (Table S1). High levels of *pqsE* were expected as a consequence of IPTG induction. The *nadA* gene, involved in the synthesis of the NAD precursor quinolinic acid (47), is located upstream from *pqsR* in the same orientation as *pqsE* (48). Deletion of the *phnAB-pqsR* gene locus relocates *nadA* immediately downstream from *pqsE* in the ΔQS-Eind strain (Fig. 1A), hence *nadA* overexpression under the *pqsE*-expressing condition is likely due to transcriptional read-through from the IPTG-inducible promoter upstream from *pqsE*. The divergent genes PA2827 and PA2828 code for the methionine sulfoxide reductase MsrB, involved in *in vitro* oxidative stress tolerance (49), and a still uncharacterized putative aminotransferase, respectively. RT-qPCR analyses confirmed that PA2827 is upregulated by *pqsE* expression, while the expression of PA2828 is not significantly affected in response to PqsE (Fig. 2A). In line with this evidence, PA2827 expression was altered in the RNA-seq analysis upon IPTG provision in both the ΔQS-Eind(pHERD30T) and ΔQS-Eind(pHERD-*rhlR*) strains, with FC values of 2.50 and 3.15 relative to the baseline condition, respectively. Conversely, the expression of PA2828 appeared to be altered only in the ΔQS-Eind(pHERD30T) strain, with a FC value close to the cutoff (2.00), but not in the ΔQS-Eind(pHERD-*rhlR*) genetic background (Table S1). Overall, PA2827 seems to be the only gene whose expression is specifically and significantly regulated by PqsE in the QS-deficient genetic background ΔQS-Eind when *rhlR* is not expressed.

Interestingly, we previously reported a negative effect exerted by PqsE on P*pqsA* activity in a PAO1 *rhlR* mutant strain (14), while in this study, the *pqsB*, *pqsC*, and *pqsD* genes (still present and under the control of P*pqsA* in ΔQS-Eind [Fig. 1A]) were not identified as differentially regulated by *pqsE* expression in the ΔQS-Eind(pHERD30T) genetic background. We reasoned that this could be due to the lack of P*pqsA* activation caused by *pqsR* deletion in the tested strains, which does not allow for a possible repressive effect exerted by PqsE on P*pqsA*. To investigate this issue, the activity of a transcriptional fusion between the P*pqsA* promoter region and the *luxCDABE* operon for bioluminescence emission (P*pqsA*::*lux*) was monitored in the *pqsR*-deficient ΔQS-Eind(pHERD-*rhlR*) strain and the *pqsR*-proficient Δ4AQΔ*rhl*Δ*las*(pHERD-*rhlR*) strain (Fig. 1A), both grown in LB supplemented with C_4_-HSL and PQS, in the presence of IPTG and l-arabinose in different combinations. As shown in Fig. 2B, light emission from the ΔQS-Eind(pHERD-*rhlR*) strain was not affected by IPTG and/or l-arabinose provision. As expected, the P*pqsA*::*lux* transcriptional fusion was significantly more active in Δ4AQΔ*rhl*Δ*las*(pHERD-*rhlR*) relative to that in ΔQS-Eind(pHERD-*rhlR*) and, in this *pqsR*-proficient background, *pqsE* expression caused a strong repression of P*pqsA* activity independent of RhlR, in accordance with previous data (14). In the presence of PqsR, P*pqsA* activity was also reduced by expression of *rhlR*, in agreement with the negative role exerted by RhlR on the *pqs* QS system (38–41). The repressive effect exerted by both PqsE and RhlR on P*pqsA* activity led to complete abrogation of bioluminescence emission when *pqsE* and *rhlR* were simultaneously expressed.

Overall, while PqsE seems to exert a mild regulatory activity on a single transcription unit (i.e., PA2827) in the ΔQS-Eind(pHERD30T) strain when *rhlR* is not expressed, PqsE likely alters the expression of additional genes (e.g., the *pqsABCDE-phnAB* operon) independently of RhlR in PqsR-proficient P. aeruginosa strains.

### (ii) PqsE expands the RhlR regulon and differentially modulates distinct subsets of RhlR-controlled genes.

The expression of 201 and 393 genes was altered upon induction of RhlR alone and in combination with PqsE, respectively (Table S1).

As expected, *rhlR* was among the 201 genes found to be differentially regulated in the ΔQS-Eind(pHERD-*rhlR*) strain grown in the presence of l-arabinose and C_4_-HSL compared to the ΔQS-Eind(pHERD30T) strain grown under the same conditions. Three out of the remaining 200 genes (i.e., PA2384, PA2755, and PA4135) showed FC values close to the cutoff and were not identified as differentially regulated by simultaneous expression of *pqsE* and *rhlR* in the ΔQS-Eind(pHERD-*rhlR*) strain. In contrast, the remaining 197 RhlR-controlled genes were also identified as differentially regulated in the ΔQS-Eind(pHERD-*rhlR*) background expressing both *pqsE* and *rhlR*, indicating that these 197 genes represent the RhlR regulon in the absence of PqsE.

The 393 genes whose expression was affected by simultaneous expression of *rhlR* and *pqsE* include the 197 genes regulated by RhlR alone, *rhlR* and *pqsE* (whose expression was induced by l-arabinose and IPTG, respectively), *nadA* and PA2827 (whose expression was altered also in response to PqsE alone), and 192 additional genes. When excluding *rhlR*, *pqsE*, *nadA*, and PA2827 from this list, 389 genes likely constitute the RhlR regulon in the presence of PqsE. Thus, while PqsE has a limited effect on the transcriptional profile of a P. aeruginosa strain lacking RhlR, PqsE becomes an important regulatory element in the presence of this QS receptor, significantly expanding the RhlR regulon from 197 to 389 genes.

**FIG 3:**
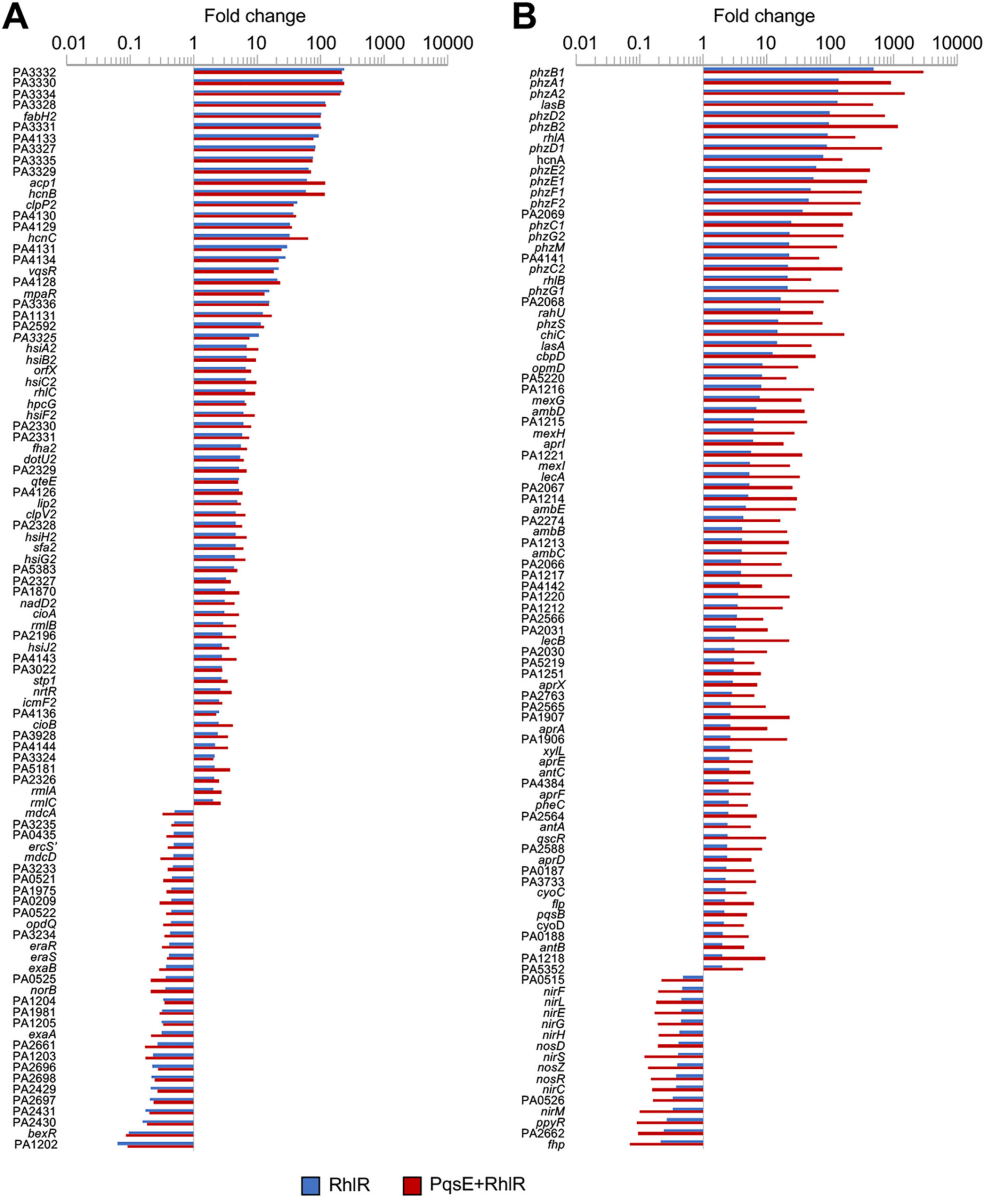
Differential impact of PqsE on the genes controlled by RhlR alone. Histograms reporting the fold change (FC) of the RhlR-controlled class I (A) and class II (B) genes determined by RNA-seq in the ΔQS-Eind(pHERD-*rhlR*) strain grown in LB supplemented with 0.1% (wt/vol) L-arabinose and 10 μM C_4_-HSL (*rhlR*-expressing condition, RhlR, blue bars), and in the ΔQS-Eind(pHERD-*rhlR*) strain grown in LB supplemented with 0.1% (wt/vol) L-arabinose, 10 μM C_4_-HSL, and 500 μM IPTG (*pqsE*-*rhlR*-expressing condition, PqsE+RhlR, red bars), relative to that in the ΔQS-Eind(pHERD30T) strain grown in LB supplemented with 0.1% (wt/vol) L-arabinose and 10 μM C_4_-HSL (baseline condition). Gene names and PA numbers are from the Pseudomonas Genome Database (102).

Even more interestingly, 98 of the 197 genes controlled by RhlR alone showed a similar FC in the RhlR versus baseline and PqsE + RhlR versus baseline comparisons, indicating that PqsE has no impact on the expression of these genes (Fig. 3A and Table S1). Conversely, 99 of the 197 RhlR-controlled genes were even more strongly affected in the presence of both RhlR and PqsE (Fig. 3B and Table S1). The 389 RhlR-controlled genes were tentatively classified into three different classes based on their PqsE dependency: (i) class I, 98 genes whose expression was altered in response to RhlR and not influenced by PqsE (Fig. 3A); (ii) class II, 99 genes whose expression was altered in response to RhlR and even more affected in the presence of both RhlR and PqsE (Fig. 3B); and (iii) class III, 192 genes whose expression was affected exclusively when RhlR and PqsE were present simultaneously. Each class contains both up- and downregulated genes (Fig. 3 and Table S1).

Many of the virulence factor genes already known to be positively controlled by RhlR and/or PqsE were identified as activated class II genes, including those required for pyocyanin and rhamnolipid synthesis and those coding for the LasA, AprA, and AprX proteases, LasB elastase, LecA and LecB lectins, ChiC chitinase, CbpD monooxygenase, MexGHI-OpmD efflux pump, and transcriptional regulators of the virulence determinants VqsR, QscR, and MpaR (Fig. 3B and Table S1) (50–65).

Interestingly, some genes involved in P. aeruginosa pathogenicity were also found among the upregulated class I genes, including those coding for elements of the type 6 secretion system Hcp1-Secretion Island II, which promotes internalization of P. aeruginosa into host epithelial cells (66); the *clpP2* gene, coding for a peptidase required for the formation of structured microcolonies and their subsequent development into mature biofilms (67); and the *rmlA*, *rmlB*, and *rmlC* genes, involved in the synthesis of the rhamnolipid precursor dTDP-l-rhamnose and the release of extracellular DNA, an important factor in the formation of antibiotic-resistant biofilms (Fig. 3A and Table S1) (68–71).

Concerning the class III virulence genes, these were mostly downregulated by RhlR and PqsE, and include the genes responsible for the regulation and synthesis of the siderophores pyoverdine (*pvd* genes) (72) and pyochelin (*pch* genes) (73) (Table S1).

Regarding the subdivision of the RhlR-controlled genes into the three aforementioned classes, we clarify that differentially expressed genes were classified as class I or class II genes based on the ratio between the FC obtained when comparing the PqsE + RhlR condition to the baseline relative to that obtained when comparing the RhlR-alone condition to the baseline. Genes with FC ratios between 0.69 and 1.99 and between 2 and 12.69 were classified as class I and class II genes, respectively. This cutoff was also chosen based on the evidence that class II genes, but not class I genes, were differentially regulated in the ΔQS-Eind strain producing PqsE + RhlR relative to the same strain producing RhlR alone (data not shown). Nevertheless, the classification of some genes into class I or class II was arbitrary, as the ability of PqsE to stimulate RhlR regulatory activity has the characteristics of a continuum rather than an all-or-nothing phenomenon. That said, the effect of PqsE was extremely pronounced on some RhlR-controlled genes, such as *phzA2* (FC RhlR versus baseline = 133.13, FC PqsE + RhlR versus baseline = 1,485.34; ratio = 11.16) and *chiC* (FC RhlR versus baseline = 14.71, FC PqsE + RhlR versus baseline = 166.62; ratio = 11.03), while it was apparently absent for others, including *qteE* (FC RhlR versus baseline = 5.14, FC PqsE+RhlR versus baseline = 5.04; ratio = 0.98) and *bexR* (FC RhlR versus baseline = −10.61 and FC PqsE + RhlR versus baseline = −11.71; ratio = 1.10).

**FIG 4:**
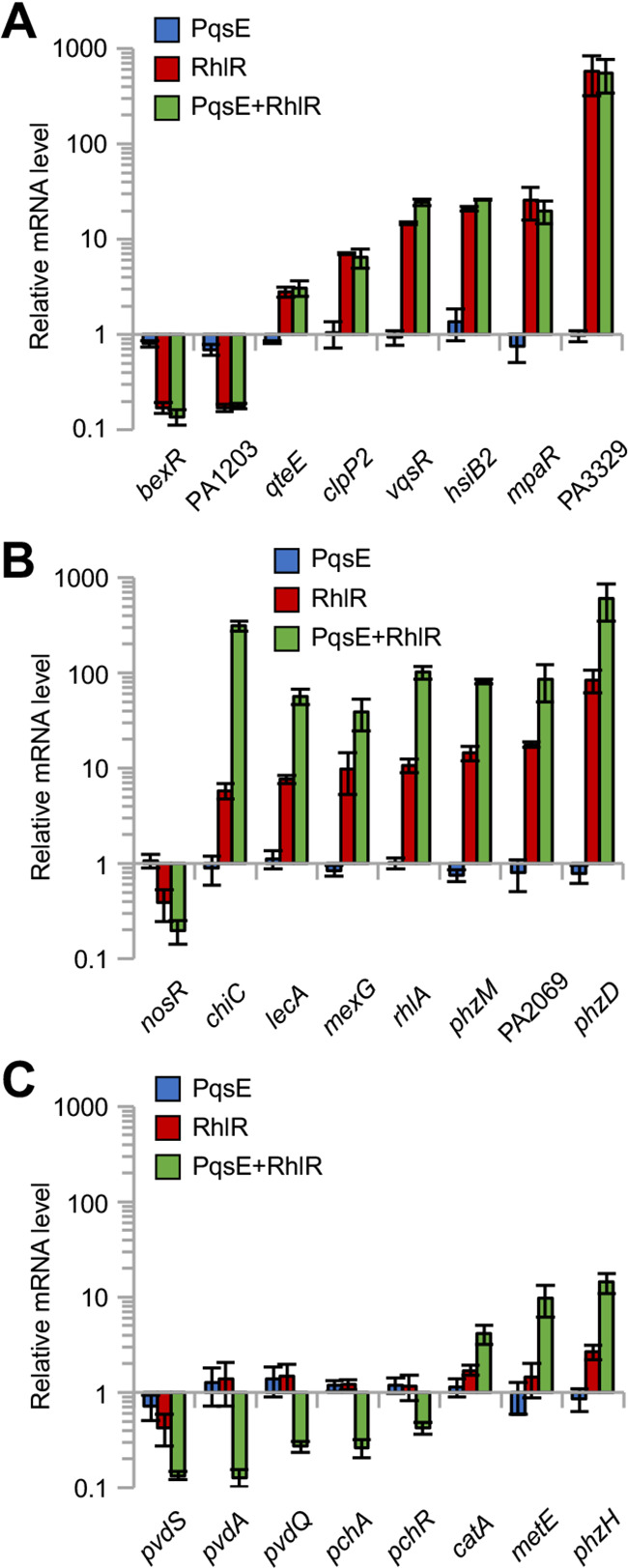
RT-qPCR analyses that corroborate the RNA-seq data. Histograms reporting the relative level of the indicated class I (A), class II (B), and class III (C) mRNAs measured by RT-qPCR in the ΔQS-Eind(pHERD30T) strain grown in LB supplemented with 0.1% (wt/vol) L-arabinose, 10 μM C_4_-HSL, and 500 μM IPTG (*pqsE*-expressing condition, PqsE, blue bars), ΔQS-Eind(pHERD-*rhlR*) strain grown in LB supplemented with 0.1% (wt/vol) L-arabinose and 10 μM C_4_-HSL (*rhlR*-expressing condition, RhlR, red bars), and ΔQS-Eind(pHERD-*rhlR*) strain grown in LB supplemented with 0.1% (wt/vol) L-arabinose, 10 μM C_4_-HSL, and 500 μM IPTG (*pqsE*-*rhlR*-expressing condition, PqsE + RhlR, green bars), relative to that in the ΔQS-Eind(pHERD30T) strain grown in LB supplemented with 0.1% (wt/vol) L-arabinose and 10 μM C_4_-HSL (baseline condition). The average of three independent experiments is reported with SD. For panels A and B, differences in the relative mRNA level with respect to the baseline condition were significant for all tested genes in both the *rhlR-* and *pqsE*-*rhlR*-expressing conditions (*P* < 0.05). For panel C, differences in the relative mRNA level with respect to the baseline condition were significant for all tested genes in the *pqsE*-*rhlR*-expressing condition, and for the *pvdS* and *phzH* genes in the *rhlR*-expressing condition (*P* < 0.05). Please note that this analysis does not allow discrimination between *phzD1* and *phzD2*, as these genes share 100% sequence identity (103). Hence, here we refer to *phzD* to indicate the mRNA levels of both *phzD1* and *phzD2*.

Differential expression of selected genes was validated by RT-qPCR analyses. As shown in Fig. 4A, the expression of the class I genes *bexR*, *PA1203*, *qteE*, *clpP2*, *vqsR*, *hsiB2*, *mpaR*, and *PA3329* was confirmed to be regulated by RhlR independently of PqsE (Fig. 4A). The expression of the class II genes *nosR*, *chiC*, *lecA*, *mexG*, *rhlA*, *phzM*, *PA2069*, and *phzD* was altered in the RhlR-producing strain, and the effect of RhlR was increased by the expression of *pqsE* (Fig. 4B). Finally, concerning class III genes, the expression of *pvdA*, *pvdQ*, *pchA*, *pchR*, *catA*, and *metE* was affected only when both *pqsE* and *rhlR* were expressed (Fig. 4C). The results obtained for *pvdS* and *phzH* were borderline between those of class II and class III genes (Fig. 4C), suggesting that the classification based on RNA-seq data could be equivocal for few genes. Nevertheless, RT-qPCR analyses confirmed the classification into class I, II, or III for almost all the selected RhlR-dependent genes.

### The C_4_-HSL signal molecule is strictly required for RhlR regulatory activity.

The RNA-seq analysis performed in this study was conducted in the presence of exogenously provided C_4_-HSL. Previous studies performed in P. aeruginosa PA14 proposed that PqsE can stimulate RhlR in promoting the expression of some target genes by producing a still-unidentified diffusible signal molecule capable of activating RhlR in a C_4_-HSL-independent manner (15, 31). However, this notion has been contradicted in a more recent study which was also performed in the PA14 strain (32).

To investigate whether C_4_-HSL could be, at least in part, dispensable for RhlR-dependent gene expression in the presence of PqsE in P. aeruginosa PAO1, we monitored the activity of transcriptional fusions between the promoter region of selected class I (i.e., *vqsR* and *hsiA2*), class II (i.e., *rhlA* and *mexG*), and class III (i.e., *pvdS* and *pchR*) genes and the *luxCDABE* operon in the ΔQS-Eind(pHERD30T) and ΔQS-Eind(pHERD-*rhlR*) strains, both grown in LB supplemented with different combinations of IPTG, l-arabinose, and C_4_-HSL.

**FIG 5:**
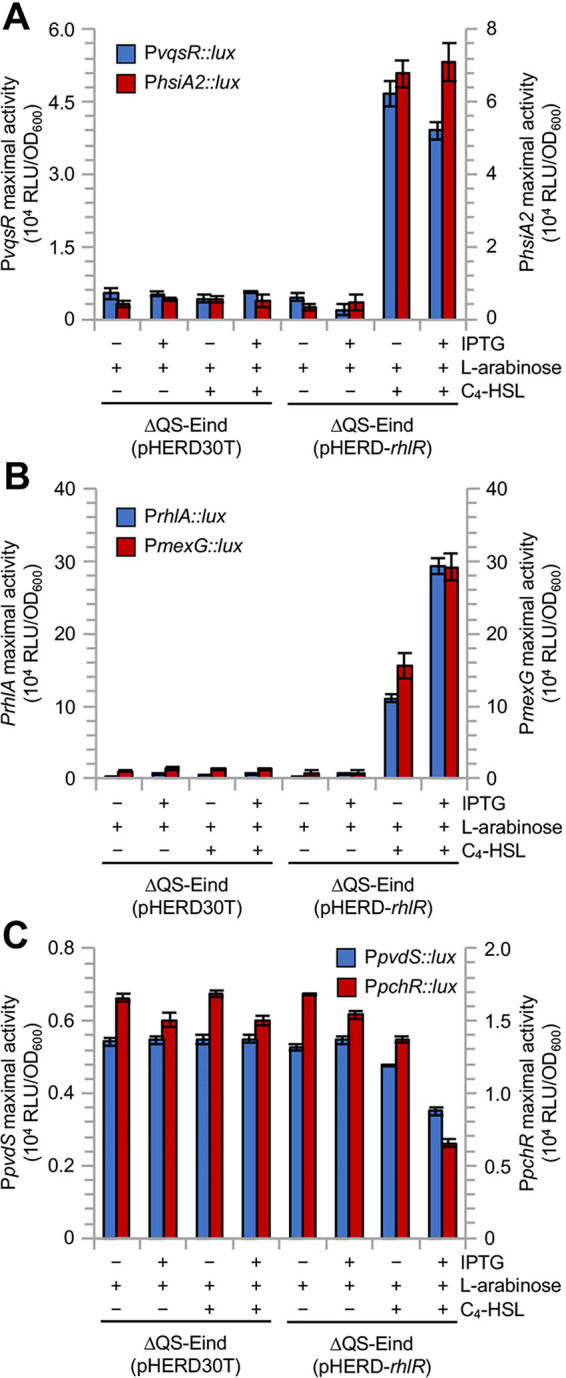
Impact of PqsE, RhlR, and C_4_-HSL on class I, II, and III promoters. Histograms reporting the maximum activity of the P*vqsR* (blue bars) and P*hsiA2* (red bars) class I promoters (A), P*rhlA* (blue bars) and P*mexG* (red bars) class II promoters (B), and P*pvdS* (blue bars) and P*pchR* (red bars) class III promoters (C), measured in the ΔQS-Eind(pHERD30T) and ΔQS-Eind(pHERD-*rhlR*) strains grown in LB supplemented (+) or not (–) with 500 μM IPTG, 0.1% (wt/vol) L-arabinose, and 10 μM C_4_-HSL. RLU, relative light units. The average of three independent experiments is reported with SD. For panels A and B, differences in the maximum promoter activity in the ΔQS-Eind(pHERD-*rhlR*) strain in response to C_4_-HSL alone, and to C_4_-HSL plus IPTG, were significant with respect to the same strain grown in the absence of C_4_-HSL and IPTG (*P* < 0.001). For panel C, differences in the maximum promoter activity in the ΔQS-Eind(pHERD-*rhlR*) strain in response to C_4_-HSL plus IPTG were significant with respect to the same strain grown in the absence of C_4_-HSL and IPTG (*P* < 0.05).

In accordance with transcriptomic and RT-qPCR data, the P*vqsR* and P*hsiA2* promoters were induced only in *rhlR*-expressing conditions, and *pqsE* expression did not affect their activity (Fig. 5A). The activity of P*rhlA* and P*mexG* increased in the presence of RhlR alone and reached the maximal level when both *rhlR* and *pqsE* were expressed (Fig. 5B). Conversely, the P*pvdS* and P*pchR* promoters appeared to be downregulated only upon expression of both *rhlR* and *pqsE* (Fig. 5C). Notably, no alteration of promoter activity was observed in the absence of C_4_-HSL for all genes (Fig. 5). Thus, besides further validating the RNA-seq data, this analysis demonstrates that the ability of RhlR to control the tested promoters strictly requires its cognate signal molecule C_4_-HSL, irrespective of the presence or absence of PqsE.

To further examine this issue, we tested the ability of cell-free supernatants from wild-type PAO1* (ATCC 15692) and its isogenic mutants with *rhlI*, *pqsE*, or both *rhlI* and *pqsE* deletions, to promote P*rhlA* activity in a P. aeruginosa PAO1* genetic background with all the QS genes of the *las*, *rhl*, and *pqs* QS systems (i.e., *lasI*, *rsaL*, *lasR*, *rhlI*, *rhlR*, *pqsABCDE-phnAB*, *pqsR*, *pqsH*, and *pqsL*) deleted, herein named the ΔQS strain. In addition, we also tested cell-free supernatants collected from cultures of the ΔQS strain carrying the pUCP18 empty vector or pUCP18 derivatives for constitutive expression of *rhlI* or *pqsE.* In our study, P*rhlA* activity increased only in the presence of cell-free supernatants collected from the C_4_-HSL producing strains, while supernatants from *pqsE*-expressing cultures failed to promote P*rhlA* activity in the absence of the RhlR cognate signal molecule C_4_-HSL (Fig. S3A). Similar results were obtained when the activity of the P*rhlA* promoter was tested in a different mutant strain with multiple deletions in *lasI*, *lasR*, *rhlI*, *rhlR*, and *pqsABCDE* (Fig. S3B), which reproduces the QS-defective genetic background used in a previous study (15).

Overall, our data indicate that the stimulating activity exerted by PqsE on RhlR strictly requires C_4_-HSL, and that PqsE does not produce a secreted molecule which can activate RhlR in the absence of C_4_-HSL, at least in P. aeruginosa PAO1.

### Further investigation revealed the existence of genes activated by RhlR and repressed by PqsE.

One of the most intriguing findings of the RNA-seq analysis was the differential ability of PqsE to affect RhlR regulatory activity towards distinct subsets of RhlR-controlled genes. Additional data analyses and experiments were conducted to delve into this issue.

**FIG 6:**
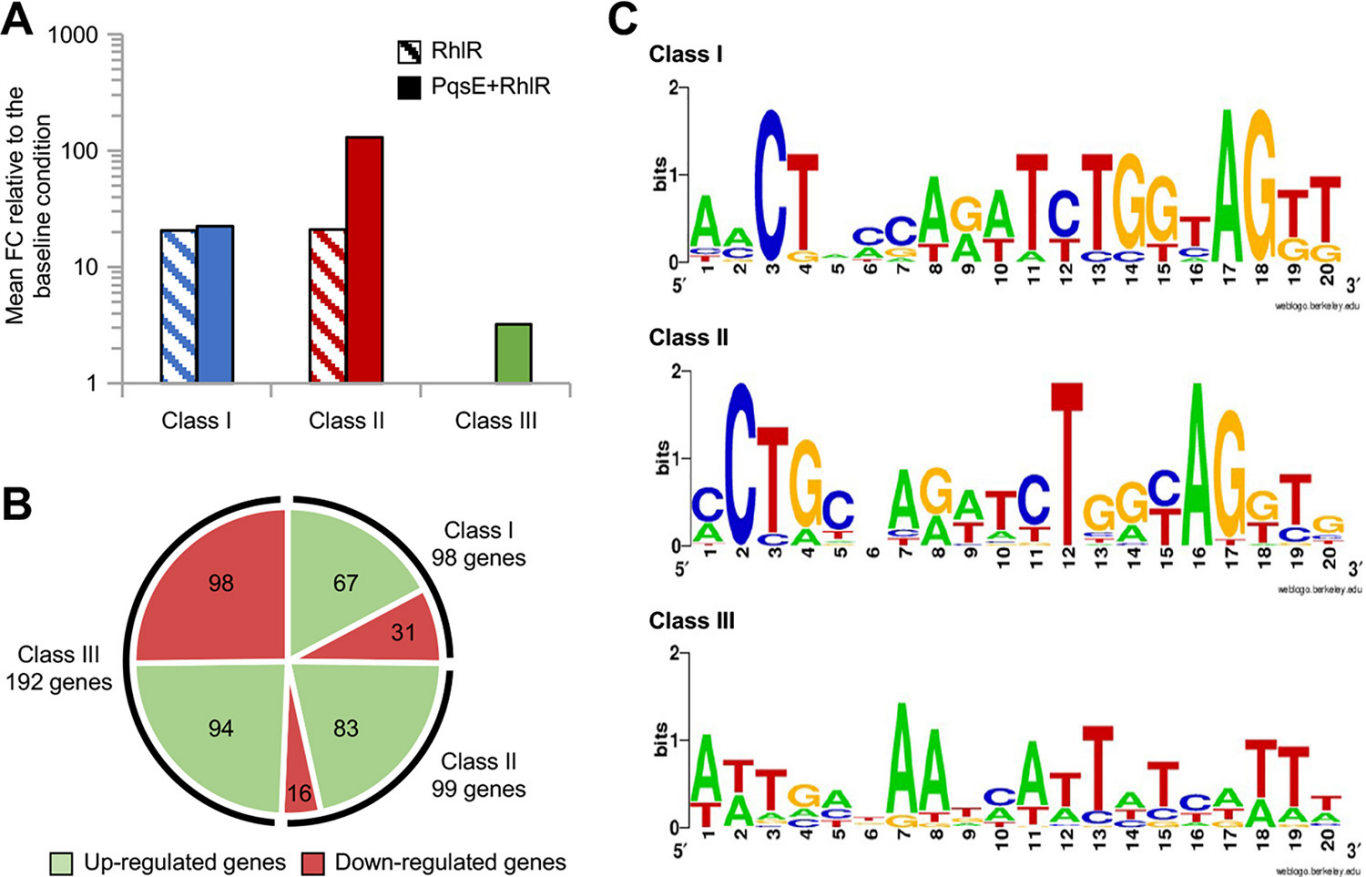
Additional analyses of class I, II, and III genes. (A) Histogram reporting the average FCs of class I (blue bars), class II (red bars), and class III (green bars) genes in RNA-seq comparisons between the ΔQS-Eind(pHERD-*rhlR*) strain grown in LB supplemented with 0.1% (wt/vol) L-arabinose and 10 μM C_4_-HSL (*rhlR*-expressing condition, RhlR, striped bars) and the ΔQS-Eind(pHERD-*rhlR*) strain grown in LB supplemented with 0.1% (wt/vol) L-arabinose, 10 μM C_4_-HSL, and 500 μM IPTG (*pqsE*-*rhlR*-expressing condition, PqsE + RhlR, full bars), relative to that of the ΔQS-Eind(pHERD30T) strain grown in LB supplemented with 0.1% (wt/vol) L-arabinose and 10 μM C_4_-HSL (baseline condition). (B) Pie chart reporting the number of upregulated (green sections) or downregulated (red sections) genes belonging to classes I, II, and III. (C) Consensus sequences identified by the pattern discovery algorithm CONSENSUS (74) on the promoter regions of all transcriptional units containing only class I, II, or III genes.

First, the mean FC values of the 389 RhlR-controlled genes grouped by classes were calculated and compared (Fig. 6A). Results showed that the mean FC values of class I genes in *pqsE*-expressing conditions and those where *pqsE* was not expressed (22.44 and 20.63, respectively), were comparable to the mean FC value of class II genes under the condition in which *rhlR* only was expressed (21.02). Conversely, the mean FC value of class II genes was much higher (129.04) when both *rhlR* and *pqsE* genes were expressed (Fig. 6A). This evidence underlies the ability of PqsE to stimulate RhlR activity to regulate class II genes and indicates that no correlation exists between the extent of RhlR-dependent gene regulation and the ability of PqsE to stimulate RhlR functionality. In contrast, the mean FC value of class III genes was much lower (3.21 [Fig. 6A]), indicating that RhlR and PqsE exert milder regulatory activity on the expression of these genes.

Differences between the class I and class II genes relative to the class III genes were observed when comparing the distribution of activated or repressed genes and when searching for putative RhlR-binding sites on their promoter regions. In fact, the majority of class I and class II genes were upregulated following the expression of *rhlR* alone or in combination with *pqsE*, in accordance with the common notion that RhlR mainly acts as a transcriptional activator (Fig. 6B). Conversely, the distribution of up- and downregulated genes was almost equal for class III genes (Fig. 6B), suggesting that a relevant proportion of these genes could be indirectly controlled by RhlR via ancillary regulators.

Concerning the presence of putative RhlR-binding sequences on the promoter regions of class I, II, and III genes, these were investigated by an unbiased *in silico* analysis based on the pattern discovery algorithm CONSENSUS (74). The analysis was performed on the 500-bp regions upstream from all transcriptional units containing class I (*n* = 41), class II (*n* = 36), or class III (*n* = 125) genes only. A sequence pattern containing the CT(N_12_)AG motif, similar to the one previously proposed for RhlR binding (75), was identified in 9 of 41 and in 18 of 36 class I and class II promoter sequences, respectively, while a different consensus sequence was identified in 36 out of 125 class III promoter regions (Fig. 6C). The matrices generated with CONSENSUS for class I and class II genes were used as queries to retrieve putative RhlR-binding sites in the 500-bp upstream regions of all the class I, II, and III transcriptional units by means of an *in silico* analysis performed with PATSER (74). The latter analysis revealed that putative RhlR-binding sites can be found in 10 out of 41 (24.4%) promoters of class I transcriptional units, 18 of 36 (50%) promoters of class II transcriptional units, and 14 of 125 (11.2%) promoters of class III transcriptional units. Interestingly, more than 90% of the putative RhlR-binding sites identified on class I, class II, and class III promoters were retrieved using either of the matrices generated by CONSENSUS for class I or class II promoters, indicating that no clear difference exists between the consensus sequences for RhlR-binding identified on the promoter regions of class I and class II genes. This analysis supports the hypothesis that a high proportion of class III genes might be controlled by RhlR-dependent ancillary regulators.

**FIG 7:**
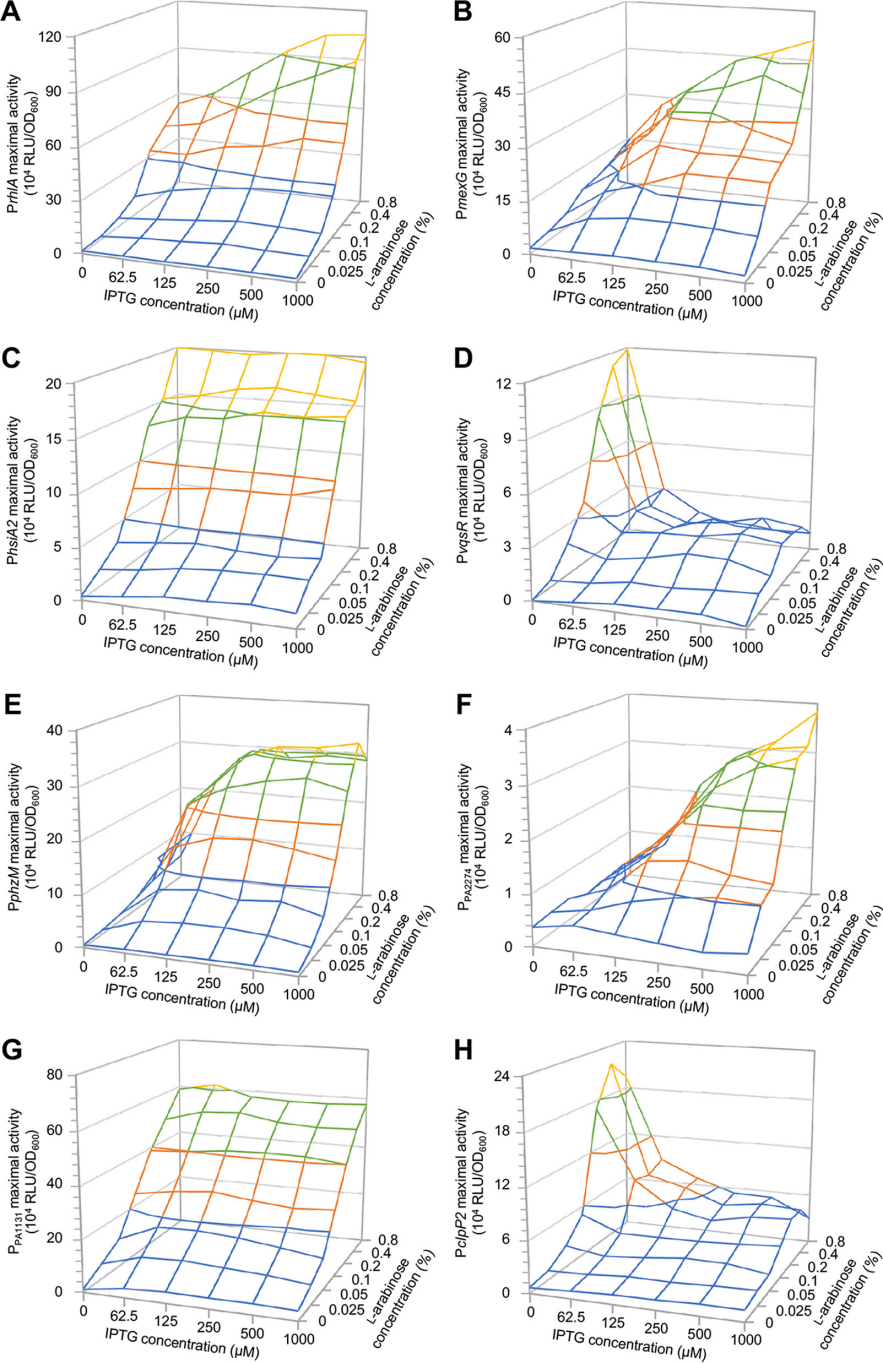
Response of class I and class II promoters to different PqsE and RhlR levels. Histograms reporting the maximum activity of the P*rhlA*::*lux* (A), P*mexG*::*lux* (B), P*hsiA2*::*lux* (C), P*vqsR*::*lux* (D), P*phzM*::*lux* (E), PPA2274::*lux* (F), PPA1131::*lux* (G), and P*clpP2*::*lux* (H) transcriptional fusions in the ΔQS-Eind(pHERD-*rhlR*) strain grown in LB supplemented with 10 μM C_4_-HSL and the indicated concentrations of IPTG and L-arabinose. RLU, relative light units. For each promoter, representative values from three independent experiments are shown.

To further investigate the differential impacts of RhlR and PqsE on class I and class II genes, the activity of selected class I and class II promoters was monitored in the ΔQS-Eind(pHERD-*rhlR*) strain grown in the presence of C_4_-HSL and different concentrations of IPTG and l-arabinose by using *lux*-based transcriptional fusions. As expected, the class II promoters P*rhlA* and P*mexG* were activated by l-arabinose alone, and their activity further increased when both l-arabinose and IPTG were present (Fig. 7A and B), while the activity of the class I promoter P*hsiA2* paralleled the increase in l-arabinose concentration, and hence that of *rhlR* expression, and was not affected by IPTG-dependent expression of *pqsE* (Fig. 7C). Surprisingly, the P*vqsR* class I promoter showed a different activation pattern in response to RhlR and PqsE relative to P*hsiA2*. Indeed, the activity of P*vqsR* was highly enhanced in response to l-arabinose alone, while concomitant addition of IPTG counteracted its RhlR-dependent activation (Fig. 7D). To corroborate these data, additional class I and class II promoters were investigated. P*phzM* and PPA2274 behaved as expected for promoters controlling class II genes, as they were activated by RhlR and even further stimulated by PqsE in the presence of RhlR (Fig. 7E and F). The class I promoter PPA1131 was activated by RhlR and insensitive to PqsE (Fig. 7G), similarly to what was observed for P*hsiA2*, while the class I promoter P*clpP2* was activated by RhlR and repressed by concomitant expression of *pqsE* (Fig. 7H), resembling the activation pattern of P*vqsR*.

It is notable that the activities of both P*vqsR* and P*clpP2* were comparable when the ΔQS-Eind(pHERD-*rhlR*) strains carrying the P*vqsR*::*lux* or P*clpP2*::*lux* fusions were grown in the presence of the *rhlR* and *pqsE* inducers at the same concentrations used in the RNA-seq analysis (i.e., 0.1% [wt/vol] l-arabinose alone, or 0.1% [wt/vol] l-arabinose plus 500 μM IPTG). It seems that limited activation of P*vqsR* and P*clpP2* when RhlR is induced with 0.1% (wt/vol) l-arabinose does not allow appreciation of the repressive effect exerted by PqsE on these promoters. Conversely, PqsE repression on P*vqsR* and P*clpP2* becomes evident at l-arabinose concentrations of  ≥0.2% (wt/vol), i.e., when higher levels of RhlR can strongly stimulate P*vqsR* and P*clpP2* activity.

Overall, these analyses confirmed that class II genes are controlled by RhlR and are sensitive to PqsE levels when RhlR is present, and highlight important differences with respect to PqsE sensitivity among class I genes. Indeed, while some genes assigned to class I are actually insensitive to PqsE levels, such as *hsiA2* and *PA1131*, other genes previously assigned to class I appear to be activated by RhlR and repressed by PqsE, such as *vqsR* and *clpP2*; hence, these genes have been reclassified as class IV genes.

## DISCUSSION

In this study, we generated recombinant strains to untangle the regulatory roles of PqsE and RhlR in P. aeruginosa PAO1. In these genetic backgrounds, *pqsE* and *rhlR* could be independently expressed, alone or in combination, so that the production of each regulatory element did not affect the levels of the other. In this way, we managed to define the regulon controlled by PqsE in the absence of RhlR in the ΔQS-Eind genetic background, which surprisingly contains a single transcriptional unit, PA2827. This gene, whose expression is activated in response to sodium hypochlorite, encodes the sulfoxide reductase MsrB, involved in *in vitro* oxidative stress resistance and required for full virulence in the insect infection model of Drosophila melanogaster (49). Although the functional link between PqsE and MsrB remains to be determined, we can reasonably exclude a general effect of PqsE on the oxidative stress response, as other genes required for P. aeruginosa antioxidant defense were not altered by *pqsE* expression.

It should be considered that PqsE likely exerts a more pronounced RhlR-independent effect on the P. aeruginosa transcriptome in the wild-type PAO1 strain compared to that in the ΔQS-Eind mutant. Indeed, in accordance with previous data (14), in this study we confirmed that PqsE negatively regulates P*pqsA* activity independently of RhlR. This effect was not observed in the RNA-seq analysis, possibly because the P*pqsA* promoter is not active in the ΔQS-Eind genetic background due to the lack of PqsR. Considering that both the *pqsR* and *lasR* genes are deleted in the ΔQS-Eind strain, this indicates that a possible RhlR-independent repressive activity of PqsE on additional genes activated by PqsR and/or LasR could have escaped our analysis. Moreover, the possibility that PqsE can directly affect the regulatory activity of transcriptional regulators other than RhlR, possibly including PqsR and LasR, or ancillary regulators controlled by these QS receptors, cannot be excluded.

From a mechanistic point of view, the RhlR-independent control exerted by PqsE on the PA2827 gene and the *pqsABCDE-phnAB* operon could be also ascribed to its thioesterase activity. This would be in line with recent findings showing that PqsE variants unable to interact with RhlR or impaired in their catalytic activity control distinct sets of genes in P. aeruginosa PA14 (23).

Although PqsE showed a limited effect on the P. aeruginosa transcriptome in the absence of RhlR, its regulatory role was evident in an RhlR-proficient genetic background, in which PqsE production significantly expanded the RhlR regulon and modulated the expression of a subgroup of RhlR-controlled genes. Here, in more detail, we show that the RhlR-regulated genes can be classified in four different classes based on their PqsE dependency: (i) the expression of class I genes is controlled by RhlR independently of PqsE; (ii) class II genes are differentially expressed in response to RhlR and even more affected when both RhlR and PqsE are present; (iii) class III genes are differentially expressed exclusively in the presence of both RhlR and PqsE; and (iv) the expression of class IV genes is promoted by RhlR and repressed by PqsE when *rhlR* is highly expressed.

Literature data showing that PqsE increases RhlR levels (33) and/or its affinity to target promoters (23) could both justify the regulatory pattern here observed for class II genes. On the other hand, it is not clear why class I genes are not affected in response to a PqsE-dependent increase in RhlR levels and/or affinity to DNA. It would be tempting to speculate that the higher affinity of RhlR to class I relative to class II promoters could result in a saturating regulative response of class I genes to RhlR alone, thus making class I genes insensitive to the stimulation of the RhlR regulatory activity caused by PqsE. However, this hypothesis contrasts with the evidence that the mean FC values of class I genes in *pqsE*-proficient and *pqsE*-deficient conditions were not higher than those of of class II genes when *rhlR* alone was expressed, and with the similarity of the putative RhlR-binding sites identified on class I and class II promoters. The insensitivity of some class I genes to PqsE could be ascribed to a contrasting positive effect exerted by PqsE on these genes via RhlR stimulation, and a simultaneous negative effect exerted by PqsE on the same genes independently of RhlR. In this case, when both *rhlR* and *pqsE* are expressed, the PqsE negative effect could be counterbalanced by the RhlR-mediated positive regulation, enhanced by PqsE itself, resulting in an apparent PqsE insensitivity. The negative control exerted by PqsE on class I genes might be not apparent when only PqsE is present, as these genes would be not expressed in the absence of RhlR.

A negative effect exerted by PqsE on the induction of RhlR-controlled promoters is evident for class IV genes. In this case, the RhlR stimulating activity is predominant for low levels of RhlR and PqsE, while the PqsE repressing effect overcomes the RhlR-mediated positive regulation when these effectors are produced at higher levels. At present, it is not possible to determine how many genes classified as class I based on the RNA-seq data are really insensitive to PqsE (proper class I genes) or are subject to an opposite effect by RhlR and PqsE (class IV genes). Moreover, it is not possible to define whether PqsE exerts its repressive effect on class IV genes via an RhlR-dependent or RhlR-independent mechanism. In this regard, since the class IV genes *vqsR* and *clpP2* are positively regulated by LasR (76, 77), future experiments performed in a LasR-proficient genetic background could help clarify whether PqsE repression on class IV genes also occurs in the absence of RhlR, when different regulators promote their expression.

The possibility that RhlR may alternatively act as a transcriptional activator or transcriptional repressor based on its activation state should be also considered. Indeed, transcriptional regulators which switch between activating and repressing functions depending on their activity/expression level have been described. As an example, in P. fluorescens ST, the StyR response regulator acts as an activator of the styrene catabolic operon when intermediate phosphorylation levels drive its binding to high-affinity sites on the P*styA* promoter. When its phosphorylation level increases, StyR turns into a repressor of the styrene catabolic operon by binding to a low-affinity binding site on P*styA* (78–80). Dual-function transcriptional regulators have been described also among QS regulators. Indeed, the QS receptors LuxR and EsaR, from Vibrio alginolyticus and Pantoea stewartii subsp. *stewartii*, respectively, can alternatively act as activators or repressors of gene transcription based on the sequence and/or positioning of their binding sites on target promoters (81, 82). In this context, our preliminary *in silico* analysis on the promoter regions of RhlR-controlled transcriptional units did not highlight clear differences between the sequences and positioning of the putative RhlR-binding sites for class I, II, and IV genes (data not shown).

Concerning class III genes, their promoter regions may contain degenerated low-affinity RhlR-binding sites, resulting in the ability of RhlR to control their expression only when its level/activity is augmented by PqsE. This would be in line with the lower mean FC values of class III genes compared to those of class I and II genes, and with the few putative RhlR-binding sites identified on the promoter regions of class III transcriptional units. However, the latter evidence, together with the different distributions of activated/repressed class III genes compared to those of class I and II genes, would be also in line with the hypothesis that a consistent fraction of class III genes is indirectly regulated by RhlR via ancillary regulators. In this context, it is noteworthy that 27 genes coding for characterized or putative transcriptional regulators have been identified in the RhlR regulon, including *qscR*, *vqsR*, *mpaR*, *bexR*, *antR*, *pvdS*, and *pchR*.

It has to be considered that every hypothesis on the differential impact of PqsE on RhlR-controlled genes is complicated by the fact that the mechanism of action of PqsE has not been clearly defined. In this regard, we demonstrated that C_4_-HSL is essential for the regulatory activity of RhlR, consistent with recent findings obtained in the PA14 strain (23, 32), while PqsE does not seem to produce a secreted molecule able to activate RhlR in PAO1, as previously described in PA14 (15, 31).

Concerning the impact on the QS regulon of the reciprocal control exerted by RhlR on the *pqs* system, and by PqsE on RhlR activity, it is interesting to highlight that RhlR seems to limit its own regulatory activity by downregulating *pqsE* expression via P*pqsA* repression, both in the absence of PqsE and even more so when both RhlR and PqsE are present. This regulatory link implies that stimuli increasing RhlR levels would decrease *pqsE* expression, thus reducing the RhlR-stimulating activity exerted by PqsE, while stimuli reducing RhlR levels would result in increased PqsE production, thus increasing the PqsE-dependent regulatory activity of RhlR. This homeostatic control of RhlR activity is expected to differentially impact the expression of genes exclusively responsive to RhlR (class I genes) compared to that of genes whose expression is controlled by both RhlR and PqsE (class II, III, and IV genes). In fact, the expression of genes regulated by both PqsE and RhlR is expected to be robust with respect to fluctuations in RhlR levels, as the increase/decrease of this regulator could be counterbalanced by consequent adjustment of PqsE levels. On the contrary, the expression of class I genes, which are insensitive to PqsE, is expected to parallel RhlR levels. This regulatory network possibly enhances P. aeruginosa phenotypic plasticity in response to environmental fluctuations and resembles the incoherent feed-forward loop generated by LasR and RsaL in the *las* QS system. Indeed, it has been shown that genes whose expression is activated by LasR and not repressed by the LasR-controlled repressor RsaL are responsive to variations in LasR levels, while the expression of genes simultaneously activated by LasR and repressed by RsaL is robust with respect to fluctuations in LasR levels (83).

It is noteworthy that many P. aeruginosa key virulence genes are classified as class II genes, strengthening the notion that both RhlR and PqsE are relevant for P. aeruginosa pathogenicity. In accordance, both the RhlR inhibitor meta-bromo-thiolactone and the PqsE inhibitors nitrofurazone and erythromycin estolate downregulate PqsE/RhlR-dependent virulence traits in P. aeruginosa, including pyocyanin production and biofilm formation (84, 85). While some rewiring of the canonical QS regulatory cascade has been observed in P. aeruginosa clinical isolates (86–88), strains defective in the *rhl* or *pqs* systems are less frequently isolated from cystic fibrosis patients compared to *las-*deficient strains (89–91). Overall, these observations support PqsE and RhlR as promising targets for the development of antivirulence drugs reducing the pathogenic potential of P. aeruginosa.

## MATERIALS AND METHODS

### Bacteria and growth conditions.

The bacterial strains used in this study are listed in Table S2 in the supplemental material. Escherichia coli and P. aeruginosa strains were routinely grown with aeration at 37°C in lysogeny broth (LB) (92) or LB supplemented with 1.5% (wt/vol) agar. When required, LB was supplemented with 50 mM 3-(*N*-morpholino)-propanesulfonic acid (MOPS [pH 7.0]), 10 μM synthetic C_4_-HSL, 20 μM synthetic PQS, 0.1% (wt/vol) l-arabinose, and/or 500 μM IPTG. Synthetic C_4_-HSL stock solution was prepared in ethyl acetate acidified with 0.1% (vol/vol) acetic acid at 10 mM concentration. The synthetic stock solution of PQS was prepared in methanol (MeOH) at 20 mM concentration.

Unless otherwise stated, antibiotics were used at the following concentrations: E. coli, 100 μg mL^−1^ ampicillin, 10 μg mL^−1^ tetracycline (Tc), 10 μg mL^−1^ gentamicin (Gm), or 30 μg mL^−1^ chloramphenicol (Cm); P. aeruginosa, 100 μg mL^−1^ Tc, 100 μg mL^−1^ Gm, 375 μg mL^−1 ^Cm, or 400 μg mL^−1^ carbenicillin.

### Recombinant DNA techniques.

The plasmids and oligonucleotides used in this study are listed in Table S2 and Table S3, respectively. Preparation of plasmid DNA, purification of DNA fragments, restriction enzyme digestions, ligations, and transformations in E. coli DH5α or S17.1λ*pir* competent cells were performed with standard procedures (92). DNA amplification was performed by PCR using the GoTaq Polymerase (Promega, Madison, WI). FastDigest restriction enzymes were purchased from Thermo Fisher Scientific (Waltham, MA). The ligation of DNA fragments was performed using T4 DNA Ligase (Promega). Plasmids were introduced into P. aeruginosa by transformation or bi-parental conjugation using E. coli S17.1λ*pir* as the donor strain (92). All plasmids generated in this study were verified by restriction analysis and DNA sequencing, and details on their construction are given in Table S2.

### Construction of recombinant strains.

P. aeruginosa mutant strains were generated by allelic exchange using pDM4-derivative plasmids, as previously described (93, 94). The construction of pDM4-derivative plasmids is described in Table S2. Plasmids were independently introduced into P. aeruginosa strains following conjugal mating with E. coli S17.1λ*pir* as the donor strain (92). Clones with a chromosomal insertion of the pDM4-derivative plasmids were selected on LB agar plates supplemented with 375 μg mL^−1 ^Cm and 15 μg mL^−1^ nalidixic acid. Plasmid excision from the chromosome was subsequently selected on LB agar plates supplemented with 10% (wt/vol) sucrose. The resulting mutant strains were confirmed by PCR analysis.

### Measurement of QS signal molecules and pyocyanin.

Levels of 3OC_12_-HSL, C_4_-HSL, and AQ signal molecules in P. aeruginosa cell-free supernatants were determined during bacterial growth by using the reporter strains described in Table S2 and the procedures described in previous works (95–97). Briefly, P. aeruginosa cultures were grown overnight in LB at 37°C with shaking (200 rpm). Following overnight growth, bacteria were diluted to an optical density at 600 nm (OD_600_) of 0.01 in 10 mL of LB supplemented with 50 mM MOPS and grown at 37°C with shaking for 8 h. Culture supernatants were withdrawn at points of the growth curve corresponding to the highest production peaks of 3OC_12_-HSL, C_4_-HSL, and AQs, respectively (24, 98).

Quantification of 3OC_12_-HSL, C_4_-HSL, and AQs was performed by adding 5 μL of cell-free culture supernatant to 195 μL of cultures of the PA14-R3 (OD_600_ = 0.045), C_4_-HSL-Rep (OD_600_ = 0.045), and AQ-Rep strains (OD_600_ = 0.1), respectively, in 96-well, black, clear-bottomed microtiter plates. The resulting microtiter plates were incubated at 37°C. Light emission (relative light units, RLU) and cell density (OD_600_) were measured after 4 h (for 3OC_12_-HSL) or 6 h (for C_4_-HSL and AQs) of incubation using an automated luminometer-spectrophotometer plate reader Spark10M (Tecan, Mannendorf, Switzerland), and RLU were normalized to cell density (OD_600_). A calibration curve was generated by growing each reporter strain in the presence of increasing concentrations of synthetic 3OC_12_-HSL, C_4_-HSL, and PQS. The resulting dose-response curves were used to extrapolate the concentration of each signal molecule in the culture supernatants.

For the pyocyanin assay, bacteria were grown in LB supplemented with 50 mM MOPS, and with different combinations of 10 μM synthetic C_4_-HSL, 0.1% (wt/vol) l-arabinose, and/or 500 μM IPTG, as indicated in the text. Pyocyanin production was qualitatively assessed by the naked eye as blue-green pigmentation in cell-free supernatants of the resulting cultures incubated for 8 h at 37°C with shaking (late stationary phase).

### RNA extraction, genome-wide expression, and RT-qPCR analyses.

RNA was extracted from the following cultures: (i) ΔQS-Eind(pHERD30T) grown in LB supplemented with 0.1% (wt/vol) l-arabinose and 10 μM C_4_-HSL (baseline condition); (ii) ΔQS-Eind(pHERD30T) grown in LB supplemented with 0.1% (wt/vol) l-arabinose, 10 μM C_4_-HSL, and 500 μM IPTG (PqsE-alone condition); (iii) ΔQS-Eind(pHERD-*rhlR*) grown in LB supplemented with 0.1% (wt/vol) l-arabinose and 10 μM C_4_-HSL (RhlR-alone condition); (iv) ΔQS-Eind(pHERD-*rhlR*) grown in LB supplemented with 0.1% (wt/vol) l-arabinose, 10 μM C_4_-HSL, and 500 μM IPTG (PqsE + RhlR condition). For each sample, three different pools of RNA were extracted in independent experiments (biological triplicates). P. aeruginosa cultures were grown overnight in LB at 37°C with shaking (200 rpm). Following overnight growth, bacteria were diluted to an OD_600_ of 0.01 into 15 mL LB supplemented with 50 mM MOPS, 0.1% (wt/vol) l-arabinose, 10 μM C_4_-HSL, and/or 500 μM IPTG. The resulting cultures were incubated at 37°C with shaking. RNA was extracted as previously described (12) from 1 mL of each culture at an OD_600_ of 1.8 (late exponential phase of growth), at which time, in the wild-type strain PAO1, the *pqs* genes are maximally expressed (14) and the *rhl* system is also active (36). Briefly, cells were mixed with 2 mL RNA Protect Bacteria Reagent (Qiagen, Hilden, Germany), and RNA was purified using an RNeasy minikit (Qiagen) including the on-column DNase I digestion step. In addition, eluted RNA was treated for 1 h at 37°C with TURBO DNase (0.2 U per μg of RNA; Ambion, Austin, TX) and with SUPERase-In (0.4 U per μg of RNA; Ambion). DNase I was removed with the RNeasy Column purification kit (Qiagen). Purified RNA was quantified using the NanoDrop 2000 spectrophotometer (Thermo Fisher Scientific). The absence of contaminating chromosomal DNA was verified by PCR using the oligonucleotides FW*pqsB* and RV*pqsB* (Table S3).

For the RNA-seq analyses, RNA quality assessment, library preparation, sequencing, and statistical analysis of the data set were performed at the GENEWIZ Biotechnology Facility (GENEWIZ, an Azenta Life Sciences Company, Leipzig, Germany).

RNA samples were quantified using a Qubit 4.0 Fluorometer (Life Technologies, Carlsbad, CA, USA) and RNA integrity was checked with an RNA kit on an Agilent 5300 Fragment Analyzer (Agilent Technologies, Palo Alto, CA, USA). rRNA depletion was performed using a NEBNext rRNA Depletion kit (New England Biolabs [NEB], Ipswich, MA). RNA sequencing library preparation was performed using the NEBNext Ultra II RNA Library Prep kit for Illumina, following the manufacturer’s recommendations (NEB, Ipswich, MA, USA). The library preparation was not directional. Briefly, enriched RNAs were fragmented according to the manufacturers’ instructions. First-strand and second-strand cDNA were subsequently synthesized. cDNA fragments were end-repaired and adenylated at the 3′ ends, and universal adapter was ligated to cDNA fragments, followed by index addition and library enrichment with limited-cycle PCR. Sequencing libraries were validated using an NGS kit on the Agilent 5300 Fragment Analyzer (Agilent Technologies), and quantified using a Qubit 4.0 Fluorometer (Invitrogen, Carlsbad, CA).

The sequencing libraries were multiplexed and loaded onto the flow cell on the Illumina NovaSeq 6000 instrument according to the manufacturer’s instructions. The samples were sequenced using a 2 × 150 Pair-End (PE) configuration v1.5. Image analysis and base calling were conducted by the NovaSeq Control Software v1.7 on the NovaSeq instrument. Raw sequence data (.bcl files) generated from Illumina NovaSeq were converted into fastq files and de-multiplexed using the Illumina bcl2fastq program v2.20. One mismatch was allowed for index sequence identification.

After investigating the quality of the raw data, sequence reads were trimmed to remove possible adapter sequences and nucleotides with poor quality using Trimmomatic v0.36. The trimmed reads were mapped to the reference genome using the Bowtie2 aligner v2.2.6. BAM files were generated as a result of this step. Unique gene hit counts were calculated using featureCounts from the Subread package v1.5.2. Only unique reads which fell within gene regions were counted.

After extraction of gene hit counts, the gene hit counts table was used for downstream differential expression analysis. Using DESeq2, comparison of gene expression between the customer-defined groups of samples was performed. The Wald test was used to generate *P* values and log_2_-fold changes that were converted to FCs. FCs of ≥ ±2.0 with an adjusted *P* value of <0.05 were considered statistically significant.

The RNA-seq data have been deposited in the NCBI Gene Expression Omnibus database (99) and are accessible through GEO Series accession number GSE200835.

For RT-qPCR analyses, cDNA synthesis was performed from 1 μg of purified RNA using the iScript Reverse Transcription Supermix for RT-qPCR kit (Bio-Rad Laboratories, Hercules, CA). Real-time PCRs were performed using the iTaq Universal SYBR Green Supermix (Bio-Rad) and the Rotor Gene 6000 Thermocycler (Corbett Research). Gene-specific primers employed in this analysis were designed using Primer-BLAST software (www.ncbi.nlm.nih.gov/tools/primer-blast) to avoid nonspecific amplification of P. aeruginosa DNA (Table S3). 16S rRNA was chosen as the internal control to normalize the real-time PCR data in every single run and to calculate the relative FC in gene expression using the 2^–ΔΔCt^ method. Average values and standard deviations were calculated from three biological replicates.

### Promoter activity assays.

For promoter activity studies, transcriptional fusions between the promoter regions of *rhlA*, *phzM*, *mexG*, *PA2274*, *vqsR*, *hsiA2*, *clpP2*, *PA1131*, *pvdS*, *pchR*, and the *luxCDABE* operon were constructed using the miniCTX-*lux* plasmid (100), as described in Table S2. All constructs were introduced as single-copy chromosomal insertions in P. aeruginosa strains by mating with E. coli S17.1λ*pir* donors.

Bioluminescence was determined as a function of population density using an automated luminometer-spectrophotometer plate reader Spark10M (Tecan). Overnight cultures of P. aeruginosa PAO1 strains carrying chromosomal P*pqsA*::*lux* (101), P*rhlA*::*lux*, P*phzM*::*lux*; P*mexG*::*lux*, PPA2274::*lux*, P*vqsR*::*lux*, P*hsiA2*::*lux*, P*clpP2*::*lux*, PPA1131::*lux*, P*pvdS*::*lux*, and P*pchR*::*lux* fusions were diluted to an OD_600_ of 0.01 in LB supplemented with 50 mM MOPS, and with l-arabinose, C_4_-HSL, and/or IPTG in different combinations, at concentrations indicated in the text. These 200-μL cultures were grown at 37°C in 96-well, black, clear-bottomed microtiter plates. Alternatively, 30% (vol/vol) cell-free supernatants were added to cultures of P. aeruginosa ΔQS(pUCP18-*rhlR*) or Δ5(pUCP18-*rhlR*) strains harboring the P*rhlA*::*lux* fusion. To collect supernatants, following overnight growth, bacterial cultures were diluted to an OD_600_ of 0.01 into 10 mL LB supplemented with 50 mM MOPS and grown at 37°C with shaking for 8 h. Luminescence and turbidity were measured every hour to determine maximal promoter activity. Luminescence is given as RLU divided by OD_600_. The average data and standard deviations were calculated from at least three independent experiments.

### Statistical analysis.

Statistical analysis was performed with GraphPad Prism 5 software using one-way analysis of variance followed by Tukey-Kramer multiple-comparison tests. Differences with a *P* value of <0.05 were considered statistically significant.
