# Supplementary material for: PqsE Expands and Differentially Modulates the RhlR Quorum Sensing Regulon in Pseudomonas aeruginosa
Source: Microbiol Spectr. 2022 May 23;10(3):e00961-22. doi: 10.1128/spectrum.00961-22 (PMC9241726; doi:10.1128/spectrum.00961-22)
Supplement: SUPPLEMENTAL FILE 1 — Supplemental material. Download spectrum.00961-22-s001.pdf, PDF file, 1.2 MB [file spectrum.00961-22-s001.pdf]

PqsE expands and differentially modulates the RhlR quorum sensing regulon in *Pseudomonas aeruginosa*

Morgana Letizia[a], Marta Mellini[a], Alessandra Fortuna[a], Paolo Visca[a,b], Francesco Imperi[a,b], Livia Leoni[a], Giordano Rampioni[a,b]#

[a] Department of Science, University Roma Tre, Rome, Italy; [b] IRCCS Fondazione Santa Lucia, Rome, Italy.

SUPPLEMENTAL MATERIAL

Figure S1. The ΔQS-Eind strain does not produce QS signal molecules and pyocyanin

Figure S2. The L-arabinose-dependent expression of *rhlR* from the pHERD-*rhlR* plasmid restores C_4-HSL and pyocyanin production in a *P. aeruginosa* Δ*rhlR* mutant

Figure S3. PqsE does not produce a secreted molecule able to activate RhlR in the absence of C_4-HSL

Table S1. List of genes whose expression is altered upon IPTG-induced and L-arabinose-induced expression of *pqsE* and/or *rhlR* in the ΔQS-Eind genetic background

Table S2. Bacterial strains and plasmids used in this study

Table S3. Oligonucleotides used in this study

Figure S1

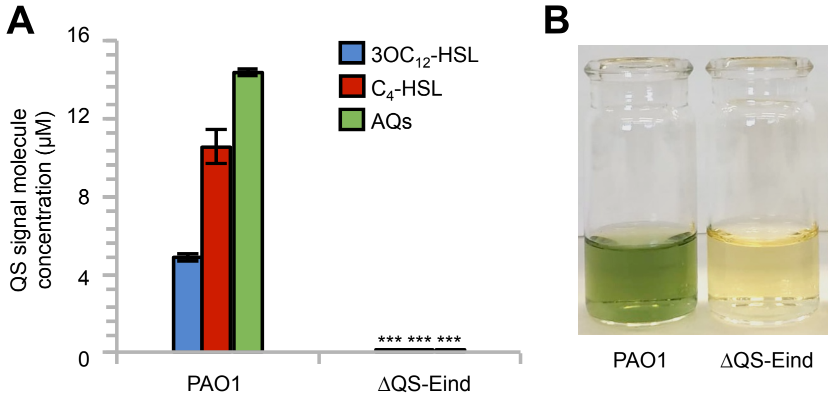

The ΔQS-Eind strain does not produce QS signal molecules and pyocyanin. (A) Histogram reporting the levels of the QS signal molecules $3OC_{12}$-HSL (blue bars), C_4-HSL (red bars), and the 2-alkyl-4-quinolones (AQs) HHQ/PQS (green bars) produced by the wild type *P. aeruginosa* PAO1 strain (PAO1) and its isogenic ΔQS-Eind mutant. The average of three independent experiments is reported with SD. ***, $P < 0.001$. **(B)** Image of cell-free supernatants from cultures of the wild type *P. aeruginosa* PAO1 Nottingham strain (PAO1) and its isogenic ΔQS-Eind mutant. A representative picture from three independent experiments is shown.

Figure S2

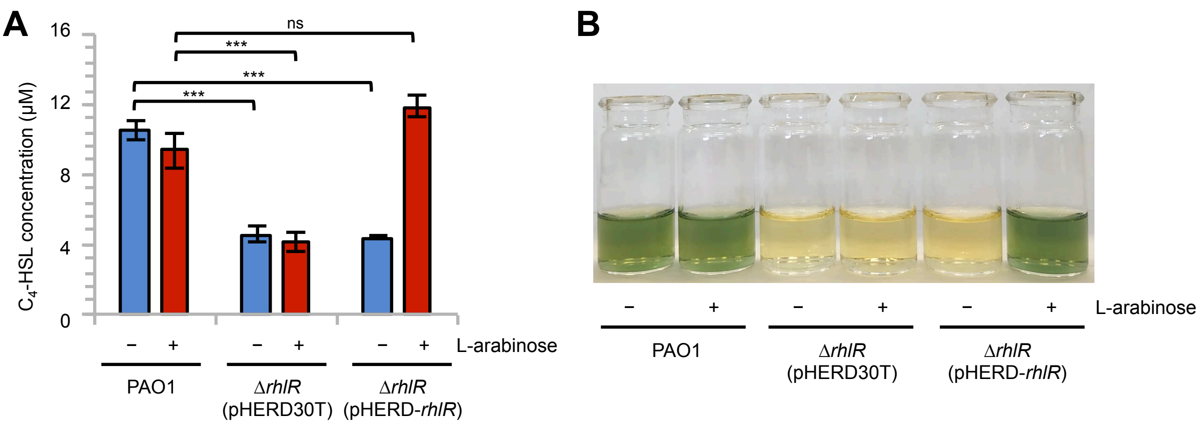

The L-arabinose-dependent expression of *rhlR* from the pHERD-*rhlR* plasmid restores C_4-HSL and pyocyanin production in a *P. aeruginosa* Δ*rhlR* mutant. (A) Histogram reporting the levels of C_4-HSL produced by the wild type *P. aeruginosa* PAO1 Nottingham strain (PAO1) and its isogenic Δ*rhlR* mutant carrying the pHERD30T empty vector or the pHERD-*rhlR* plasmid. The strains were grown in LB supplemented (+, red bars) or not (-, blue bars) with 0.1% (w/v) L-arabinose. The average of three independent experiments is reported with SD. ***, $P < 0.001$; ns, not statistically significant. **(B)** Image of cell-free supernatants from the same cultures as in **(A)**. A representative picture from three independent experiments is shown.

Figure S3

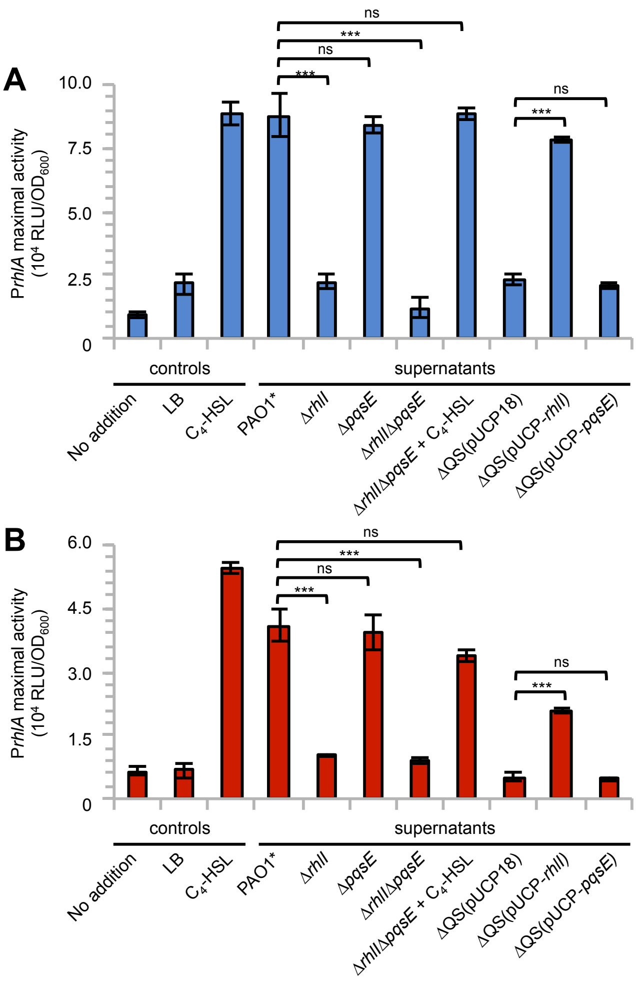

PqsE does not produce a secreted molecule able to activate RhlR in the absence of C_4-HSL. Histograms reporting the maximal activity of the P*rhlA::luxCDABE* transcriptional fusion in the (A) ΔQS mutant strain (Δ*lasI*, Δ*rsaL*, Δ*lasR*, Δ*rhlI*, Δ*rhlR*, Δ*pqsABCDE-phnAB*, Δ*pqsR*, Δ*pqsH*, Δ*pqsL*), and in (B) the Δ5 mutant strain (Δ*lasI*, Δ*lasR*, Δ*rhlI*, Δ*rhlR*, Δ*pqsE*), both carrying the pUCP-*rhlR* plasmid for constitutive expression of *rhlR*, grown in 140 µL of LB (No addition), in 200 µL of LB supplemented (C_4-HSL) or not (LB) with 10 µM C_4-HSL, or in 140 µL of LB supplemented with 60 µL [30% (v/v)] cell-free supernatants from the indicated strains. PAO1*, wild type strain of *P. aeruginosa* PAO1 ATCC 15692. The ΔQS, Δ5, Δ*rhlI*, Δ*pqsE* and Δ*rhlI*Δ*pqsE* mutants described in this figure were obtained in PAO1*. The pUCP-*rhlI* and pUCP-*pqsE* plasmids are pUCP18 derivatives for constitutive expression of *rhlI* or *pqsE*, respectively. The average of three independent experiments is reported with SD. ***, $P < 0.001$; ns, not statistically significant.

Table S1. List of genes whose expression is altered upon IPTG-induced and L-arabinose-induced expression of *pqsE* and/or *rhlR* in the ΔQS-Eind genetic background

PA number[a]	Gene name[a]	PqsE[b]	RhlR[c]	PqsE+RhlR[d]	Product name[a]
PA0024	*hemF*			-2.06	Coproporphyrinogen III oxidase, aerobic
PA0045				-2.02	Hypothetical protein
PA0050				2.48	Hypothetical protein
PA0051*∫	*phzH*			8.14	Potential phenazine-modifying enzyme
PA0052				5.16	Hypothetical protein
PA0122*∫	*rahU*		16.39	54.17	RahU
PA0123				4.35	Probable transcriptional regulator
PA0132*	*bauA*			2.43	Beta-alanine:pyruvate transaminase
PA0187*			2.33	6.29	Hypothetical protein
PA0188			2.03	5.18	Hypothetical protein
PA0208*	*mdcA*		-2.01	-3.13	Malonate decarboxylase alpha subunit
PA0209			-2.25	-3.45	Conserved hypothetical protein
PA0210	*mdcC*			-2.85	Malonate decarboxylase delta subunit
PA0211	*mdcD*		-2.11	-3.34	Malonate decarboxylase beta subunit
PA0212	*mdcE*			-3.28	Malonate decarboxylase gamma subunit
PA0213*				-2.64	Hypothetical protein
PA0214				-2.22	Probable acyl transferase
PA0236∫				2.02	Probable transcriptional regulator
PA0269*∫				3.77	Conserved Hypothetical protein
PA0270*				3.72	Hypothetical protein
PA0271*∫				3.93	Hypothetical protein
PA0272				2.42	Probable transcriptional regulator
PA0281*	*cysW*			-2.31	Sulfate transport protein CysW
PA0282	*cysT*			-2.16	Sulfate transport protein CysT
PA0283	*sbp*			-2.06	Sulfate-binding protein precursor
PA0284				-2.06	Hypothetical protein
PA0315*				2.15	Hypothetical protein
PA0366	*laoC*			2.14	LaoC
PA0430*	*metF*			2.81	5,10-methylenetetrahydrofolate reductase
PA0431*				3.08	Hypothetical protein
PA0432*	*sahH*			4.18	S-adenosyl-L-homocysteine hydrolase
PA0434				-2.41	Hypothetical protein
PA0435			-2.07	-2.71	Hypothetical protein
PA0472	*fiuI*			-2.24	FiuI
PA0480*				3.44	Probable hydrolase
PA0485				-2.08	Conserved hypothetical protein
PA0509*	*nirN*			-5.22	Probable c-type Cytochrome
PA0510*	*nirE*		-2.19	-5.81	NirE
PA0511*	*nirJ*			-4.53	Heme d1 biosynthesis protein NirJ

PA number[a]	Gene name[a]	PqsE[b]	RhlR[c]	PqsE+RhlR[d]	Product name[a]
PA0512*	*nirH*		-2.38	-5.02	NirH
PA0513*	*nirG*		-2.25	-5.13	NirG
PA0514*	*nirL*		-2.19	-5.46	Heme d1 biosynthesis protein NirL
PA0515*			-2.09	-4.49	Probable transcriptional regulator
PA0516*	*nirF*		-2.12	-5.12	Heme d1 biosynthesis protein NirF
PA0517*	*nirC*		-2.68	-6.34	Probable c-type Cytochrome precursor
PA0518*	*nirM*		-3.00	-10.07	Cytochrome c-551 precursor
PA0519*	*nirS*		-2.49	-8.36	Nitrite reductase precursor
PA0520*	*nirQ*			-2.02	Regulatory protein NirQ
PA0521*			-2.18	-3.02	Probable Cytochrome c oxidase subunit
PA0522			-2.25	-2.75	Hypothetical protein
PA0523*	*norC*			-3.48	Nitric-oxide reductase subunit C
PA0524*	*norB*		-2.82	-4.79	Nitric-oxide reductase subunit B
PA0525*			-2.78	-4.80	Probable dinitrification protein NorD
PA0526*			-2.98	-6.12	Hypothetical protein
PA0534	*pauB1*			2.21	FAD-dependent oxidoreductase
PA0545∫				-2.50	Hypothetical protein
PA0546*	*metK*			3.75	Methionine adenosyltransferase
PA0547				3.57	Probable transcriptional regulator
PA0848*∫	*ahpB*			3.47	Probable alkyl hydroperoxide reductase
PA0849*∫	*trxB2*			2.87	Thioredoxin reductase 2
PA0852*∫	*cbpD*		12.44	58.65	Chitin-binding protein CbpD precursor
PA0887	*acsA*			-3.13	acetyl-coenzyme A synthetase
PA0938*∫	*wzz2*			2.14	Wzz2
PA0997∫	*pqsB*		2.16	4.92	Homologous to beta-keto-acyl-acyl-carrier protein synthase
PA0998∫	*pqsC*			2.61	Homologous to beta-keto-acyl-acyl-carrier protein synthase
PA1000	*pqsE*	29.05		30.35	Quinolone signal response protein
PA1004	*nadA*	3.75		3.51	Quinolinate synthetase A
PA1072*	*braE*			-2.17	Branched-chain amino acid transport protein BraE
PA1073*	*braD*			-2.15	Branched-chain amino acid transport protein BraD
PA1129	*fosA*			2.36	Fosfomycin resistance protein, FosA
PA1130∫	*rhlC*		6.51	9.28	Rhamnosyltransferase 2
PA1131∫			12.32	17.08	Probable major facilitator superfamily (MFS) transporter
PA1168				6.57	Hypothetical protein
PA1202			-15.98	-11.03	Probable hydrolase
PA1203			-4.35	-5.77	Hypothetical protein
PA1204			-3.03	-2.89	NAD(P)H quinone oxidoreductase
PA1205			-3.21	-3.04	Conserved hypothetical protein
PA1211*				9.36	Hypothetical protein
PA1212*			3.53	17.90	Probable major facilitator superfamily (MFS) transporter
PA1213*			4.12	22.25	Hypothetical protein
PA1214*			5.16	29.93	Hypothetical protein
PA1215*∫			6.33	43.50	Hypothetical protein

PA number[a]	Gene name[a]	PqsE[b]	RhlR[c]	PqsE+RhlR[d]	Product name[a]
PA1216*ʃ			8.21	55.12	Hypothetical protein
PA1217*			3.95	25.20	Probable 2-isopropylmalate synthase
PA1218*ʃ			2.00	9.52	Hypothetical protein
PA1219*				8.04	Hypothetical protein
PA1220*			3.55	22.88	Hypothetical protein
PA1221*ʃ			5.68	36.14	Hypothetical protein
PA1230				2.32	Hypothetical protein
PA1245	*aprX*		2.93	7.09	AprX
PA1246	*aprD*		2.38	5.81	Alkaline protease secretion protein AprD
PA1247	*aprE*		2.58	6.03	Alkaline protease secretion protein AprE
PA1248	*aprF*		2.52	5.62	Alkaline protease secretion outer membrane protein AprF precursor
PA1249	*aprA*		2.70	10.20	Alkaline metalloproteinase precursor
PA1250	*aprI*		6.11	18.41	Alkaline proteinase inhibitor AprI
PA1251			3.03	8.10	Probable chemotaxis transducer
PA1300*	*hxuI*			-2.39	HxuI
PA1317*	*cyoA*			3.98	Cytochrome o ubiquinol oxidase subunit II
PA1318*	*cyoB*			3.61	Cytochrome o ubiquinol oxidase subunit I
PA1319*	*cyoC*		2.25	4.81	Cytochrome o ubiquinol oxidase subunit III
PA1320*	*cyoD*		2.14	4.39	Cytochrome o ubiquinol oxidase subunit IV
PA1321*	*cyoE*			3.28	Cytochrome o ubiquinol oxidase protein CyoE
PA1656ʃ	*hsiA2*		6.79	10.41	HsiA2
PA1657ʃ	*hsiB2*		6.78	9.53	HsiB2
PA1658*ʃ	*hsiC2*		6.59	9.66	HsiC2
PA1659ʃ	*hsiF2*		6.09	9.17	HsiF2
PA1660ʃ	*hsiG2*		4.42	6.48	HsiG2
PA1661	*hsiH2*		4.57	6.79	HsiH2
PA1662*ʃ	*clpV2*		4.61	6.49	ClpV2
PA1663ʃ	*sfa2*		4.56	6.09	Sfa2
PA1664ʃ	*orfX*		6.65	8.05	OrfX
PA1665*ʃ	*fha2*		5.58	6.92	Fha2
PA1666ʃ	*lip2*		4.88	5.58	Lip2
PA1667ʃ	*hsiJ2*		2.79	3.65	HsiJ2
PA1668	*dotU2*		5.37	6.17	DotU2
PA1669*ʃ	*icmF2*		2.52	2.82	IcmF2
PA1670	*stp1*		2.74	3.43	Stp1
PA1784*				2.44	Hypothetical protein
PA1837				-2.40	Hypothetical protein
PA1838	*cysI*			-2.02	Sulfite reductase
PA1869*ʃ	*acp1*		60.67	117.21	Acp1
PA1870			3.13	5.26	Hypothetical protein
PA1871*ʃ	*lasA*		14.63	50.48	LasA protease precursor
PA1872				2.78	Hypothetical protein

PA number[a]	Gene name[a]	PqsE[b]	RhlR[c]	PqsE+RhlR[d]	Product name[a]
PA1873				2.98	Hypothetical protein
PA1898	*qscR*		2.43	9.85	Quorum-sensing control repressor
PA1899∫	*phzA2*		133.13	1485.34	Probable phenazine biosynthesis protein
PA1900∫	*phzB2*		95.45	1170.18	Probable phenazine biosynthesis protein
PA1901*∫[#]	*phzC2*		21.81	156.39	Phenazine biosynthesis protein PhzC
PA1902*∫[#]	*phzD2*		97.74	729.54	Phenazine biosynthesis protein PhzD
PA1903*∫[#]	*phzE2*		60.34	418.75	Phenazine biosynthesis protein PhzE
PA1904*∫[#]	*phzF2*		46.05	299.31	Probable phenazine biosynthesis protein
PA1905*∫[#]	*phzG2*		22.93	162.86	Probable pyridoxamine 5'-phosphate oxidase
PA1906*			2.67	21.08	Hypothetical protein
PA1907*			2.71	23.11	Hypothetical protein
PA1908				2.50	Probable major facilitator superfamily (MFS) transporter
PA1927*	*metE*			10.08	5-methyltetrahydropteroyltriglutamate-homocysteine S-methyltransferase
PA1939				2.82	Hypothetical protein
PA1975			-2.24	-2.68	Hypothetical protein
PA1976	*ercS'*		-2.08	-2.57	ErcS'
PA1977				-2.06	Hypothetical protein
PA1979	*eraS*		-2.45	-2.67	Sensor kinase, EraS
PA1980	*eraR*		-2.43	-3.16	Response regulator EraR
PA1981			-3.17	-3.48	Hypothetical protein
PA1982	*exaA*		-3.22	-4.67	Quinoprotein ethanol dehydrogenase
PA1983	*exaB*		-2.73	-3.49	Cytochrome c550
PA1984	*exaC*			-2.10	NAD+ dependent aldehyde dehydrogenase ExaC
PA2028				2.00	probable transcriptional regulator
PA2030*			3.11	10.09	Hypothetical protein
PA2031*∫			3.31	10.40	Hypothetical protein
PA2065	*pcoA*			2.44	Copper resistance protein A precursor
PA2066*∫			3.96	17.14	Hypothetical protein
PA2067*∫			5.39	25.32	Probable hydrolase
PA2068*∫			16.72	78.68	Probable MFS transporter
PA2069*∫			36.82	223.90	Probable carbamoyl transferase
PA2126*	*cgrC*			-2.12	*cupA* gene regulator C, CgrC
PA2171*				2.32	Hypothetical protein
PA2193*∫	*hcnA*		77.40	155.79	Hydrogen cyanide synthase HcnA
PA2194*∫	*hcnB*		58.66	116.94	Hydrogen cyanide synthase HcnB
PA2195*∫	*hcnC*		32.41	63.66	Hydrogen cyanide synthase HcnC
PA2196*			2.83	4.67	TetR family transcriptional regulator
PA2203				-2.58	Probable amino acid permease
PA2204				-2.74	Putative binding protein component of ABC transporter
PA2252				-2.11	Probable AGCS sodium/alanine/glycine symporter
PA2260				-2.20	Hypothetical protein
PA2274*∫			4.32	16.34	Hypothetical protein

PA number[a]	Gene name[a]	PqsE[b]	RhlR[c]	PqsE+RhlR[d]	Product name[a]
PA2275				2.31	Probable alcohol dehydrogenase (Zn-dependent)
PA2297				2.18	Probable ferredoxin
PA2298*				2.07	Probable oxidoreductase
PA2299*				2.33	Probable transcriptional regulator
PA2300*∫	*chiC*		14.71	166.62	Chitinase
PA2301				2.82	Hypothetical protein
PA2302	*ambE*		4.67	28.48	AmbE
PA2303	*ambD*		6.94	39.71	AmbD
PA2304	*ambC*		4.03	20.79	AmbC
PA2305	*ambB*		4.12	21.12	AmbB
PA2326			2.11	2.51	Hypothetical protein
PA2327*			3.22	3.87	Probable permease of ABC transporter
PA2328*			4.58	5.81	Hypothetical protein
PA2329*			5.16	6.78	Probable ATP-binding component of ABC transporter
PA2330*			6.04	8.02	Hypothetical protein
PA2331*			5.79	7.42	Hypothetical protein
PA2365*	*hsiB3*			2.16	HsiB3
PA2381*				2.82	Hypothetical protein
PA2384			2.07		Hypothetical protein
PA2385	*pvdQ*			-5.04	3-oxo-C12-homoserine lactone acylase PvdQ
PA2386	*pvdA*			-6.64	L-ornithine *N5*-oxygenase
PA2389	*pvdR*			-2.34	PvdR
PA2391	*opmQ*			-2.09	Probable outer membrane protein precursor
PA2392	*pvdP*			-4.11	PvdP
PA2393				-5.05	Putative dipeptidase
PA2394	*pvdN*			-3.58	PvdN
PA2395	*pvdO*			-5.88	PvdO
PA2396	*pvdF*			-3.98	Pyoverdine synthetase F
PA2399	*pvdD*			-2.77	Pyoverdine synthetase D
PA2400	*pvdJ*			-2.58	PvdJ
PA2402				-3.69	Pyoverdine peptide synthetase
PA2411				-9.64	Probable thioesterase
PA2412				-7.95	Conserved Hypothetical protein
PA2413	*pvdH*			-7.18	L-2,4-diaminobutyrate:2-ketoglutarate 4-aminotransferase, PvdH
PA2424	*pvdL*			-3.37	PvdL
PA2425	*pvdG*			-5.49	PvdG
PA2426	*pvdS*			-6.76	Sigma factor PvdS
PA2427				-4.94	Hypothetical protein
PA2428				-2.07	Hypothetical protein
PA2429			-4.75	-3.70	Hypothetical protein
PA2430			-6.43	-5.44	Conserved Hypothetical protein
PA2431			-5.77	-4.97	Hypothetical protein

PA number[a]	Gene name[a]	PqsE[b]	RhlR[c]	PqsE+RhlR[d]	Product name[a]
PA2432	*bexR*		-10.61	-11.71	bistable expression regulator, BexR
PA2448*∫				4.34	Putative hydrolase
PA2468	*foxI*			-2.05	ECF sigma factor FoxI
PA2506				3.10	Hypothetical protein
PA2507	*catA*			3.25	Catechol 1,2-dioxygenase
PA2508	*catC*			2.72	Muconolactone delta-isomerase
PA2509*	*catB*			2.82	Muconate cycloisomerase I
PA2511*	*antR*			3.21	AntR
PA2512	*antA*		2.43	5.61	Anthranilate dioxygenase large subunit
PA2513	*antB*		2.01	4.44	Anthranilate dioxygenase small subunit
PA2514	*antC*		2.58	5.54	Anthranilate dioxygenase reductase
PA2515	*xylL*		2.66	5.82	*cis*-1,2-dihydroxycyclohexa-3,4-diene carboxylate dehydrogenase
PA2516	*xylZ*			2.74	Toluate 1,2-dioxygenase electron transfer component
PA2517	*xylY*			3.43	Toluate 1,2-dioxygenase beta subunit
PA2518	*xylX*			2.45	Toluate 1,2-dioxygenase alpha subunit
PA2563				2.21	Probable Sulfate transporter
PA2564*∫			2.52	6.96	Hypothetical protein
PA2565*∫			2.73	9.69	Hypothetical protein
PA2566*∫			3.42	8.79	Conserved hypothetical protein
PA2570*∫	*lecA*		5.40	33.07	LecA
PA2571				2.02	Probable two-component sensor
PA2588*∫			2.40	8.49	Probable transcriptional regulator
PA2589				3.72	Hypothetical protein
PA2591	*vqsR*		21.84	18.35	VqsR
PA2592∫			11.39	12.90	Probable periplasmic spermidine/putrescine-binding protein
PA2593	*qteE*		5.14	5.04	Quorum threshold expression element, QteE
PA2661			-3.70	-5.89	Hypothetical protein
PA2662			-4.15	-10.62	Conserved hypothetical protein
PA2663	*ppyR*		-3.75	-11.12	*psl* and pyoverdine operon regulator, PpyR
PA2664	*fhp*		-4.68	-14.32	Flavohemoprotein
PA2682*				2.35	Conserved Hypothetical protein
PA2696			-4.49	-3.64	Probable transcriptional regulator
PA2697			-4.88	-4.33	Hypothetical protein
PA2698			-4.61	-4.11	Probable hydrolase
PA2755			-2.06		Hypothetical protein
PA2763			2.84	6.41	Hypothetical protein
PA2787	*cpg2*			2.12	Carboxypeptidase G2 precursor
PA2788*∫				2.65	Probable chemotaxis transducer
PA2789				-2.21	Hypothetical protein
PA2827		2.50		3.15	Conserved hypothetical protein
PA2828		2.00			Probable aminotransferase
PA3022			2.77	2.84	Hypothetical protein

PA number[a]	Gene name[a]	PqsE[b]	RhlR[c]	PqsE+RhlR[d]	Product name[a]
PA3031				2.03	Hypothetical protein
PA3032*ʃ	*snr1*			6.10	Cytochrome c Snr1
PA3038*	*opdQ*		-2.28	-3.02	OpdQ
PA3049	*rmf*			2.79	Ribosome modulation factor
PA3104	*xcpP*			2.05	Secretion protein XcpP
PA3105	*xcpQ*			2.64	General secretion pathway protein D
PA3233			-2.12	-2.59	Hypothetical protein
PA3234			-2.35	-2.90	Probable sodium:solute symporter
PA3235			-2.04	-2.26	Conserved Hypothetical protein
PA3268				-2.77	Probable TonB-dependent receptor
PA3309*				-2.09	Conserved Hypothetical protein
PA3324			2.13	2.04	Probable short-chain dehydrogenase
PA3325			10.53	7.55	Conserved hypothetical protein
PA3326ʃ	*clpP2*		43.06	37.49	ClpP2
PA3327ʃ			82.40	80.29	Probable non-ribosomal peptide synthetase
PA3328*ʃ			118.61	121.42	Probable FAD-dependent monooxygenase
PA3329*ʃ			64.62	70.78	Hypothetical protein
PA3330*ʃ			222.29	234.33	Probable short chain dehydrogenase
PA3331*ʃ			98.70	102.31	Cytochrome P450
PA3332*ʃ			234.08	215.14	Conserved hypothetical protein
PA3333*ʃ	*fabH2*		101.99	100.21	3-oxoacyl-[acyl-carrier-protein] synthase III
PA3334*ʃ			213.33	202.71	Acp3
PA3335*ʃ			76.39	74.85	Hypothetical protein
PA3336*ʃ			15.50	15.33	Probable MFS transporter
PA3361*	*lecB*		3.13	22.74	Fucose-binding lectin PA-IIL
PA3362				2.00	Hypothetical protein
PA3391*	*nosR*		-2.68	-6.67	Regulatory protein NosR
PA3392*	*nosZ*		-2.55	-7.39	Nitrous-oxide reductase precursor
PA3393*	*nosD*		-2.43	-5.18	NosD protein
PA3395*	*nosY*			-2.28	NosY protein
PA3396*	*nosL*			-2.81	NosL protein
PA3409	*hasS*			-2.24	HasS
PA3410	*hasI*			-2.20	HasI
PA3450	*isfA*			-2.75	1-Cys peroxiredoxin LsfA
PA3475	*pheC*		2.52	5.04	Cyclohexadienyl dehydratase precursor
PA3477*ʃ	*rhlR*		8405.77	7231.62	Transcriptional regulator RhlR
PA3478*ʃ	*rhlB*		21.44	50.36	Rhamnosyltransferase chain B
PA3479*ʃ	*rhlA*		91.88	249.27	Rhamnosyltransferase chain A
PA3520*ʃ				5.29	Hypothetical protein
PA3573				-2.39	Probable major facilitator superfamily (MFS) transporter
PA3622*	*rpoS*			2.11	Sigma factor RpoS
PA3718*ʃ				3.20	Probable major facilitator superfamily (MFS) transporter
PA3724*ʃ	*lasB*		130.13	475.31	Elastase LasB

PA number[a]	Gene name[a]	PqsE[b]	RhlR[c]	PqsE+RhlR[d]	Product name[a]
PA3733a				3.09	Hypothetical protein
PA3734*∫			2.26	6.75	Hypothetical protein
PA3880*				-2.94	Conserved hypothetical protein
PA3909*∫	*eddB*			2.51	Hypothetical protein
PA3911				-3.07	Conserved Hypothetical protein
PA3912				-3.17	Conserved hypothetical protein
PA3913				-2.93	Probable protease
PA3928*			2.41	3.48	Hypothetical protein
PA3929*	*cioB*		2.47	4.14	Cyanide insensitive terminal oxidase
PA3930*	*cioA*		3.06	5.13	Cyanide insensitive terminal oxidase
PA4078*∫				7.51	Probable nonribosomal peptide synthetase
PA4126			5.12	5.89	Probable major facilitator superfamily (MFS) transporter
PA4127	*hpcG*		6.31	6.75	2-oxo-hept-3-ene-1,7-dioate hydratase
PA4128			20.98	23.00	Conserved hypothetical protein
PA4129			32.95	35.28	Hypothetical protein
PA4130			36.70	40.81	Probable sulfite or nitrite reductase
PA4131∫			29.57	24.10	Probable iron-sulfur protein
PA4132	*mpaR*		15.58	13.02	MpaR
PA4133			92.87	76.59	Cytochrome c oxidase subunit (*cbb3*-type)
PA4134			27.78	21.84	Hypothetical protein
PA4135			2.04		Probable transcriptional regulator
PA4136			2.50	2.25	Probable major facilitator superfamily (MFS) transporter
PA4139*				2.06	Hypothetical protein
PA4141*∫			22.61	67.68	Hypothetical protein
PA4142*∫			3.80	8.40	Probable secretion protein
PA4143*			2.77	4.72	Probable toxin transporter
PA4144*			2.16	3.49	Probable outer membrane protein precursor
PA4166				2.54	Probable acetyltransferase
PA4205*∫	*mexG*		7.80	35.23	Hypothetical protein
PA4206*∫	*mexH*		6.18	27.50	Probable Resistance-Nodulation-Cell Division (RND) efflux membrane fusion protein precursor
PA4207*∫	*mexI*		5.48	23.24	Probable Resistance-Nodulation-Cell Division (RND) efflux transporter
PA4208*∫	*opmD*		8.58	31.45	Probable outer membrane protein precursor
PA4209*∫	*phzM*		22.81	128.14	Probable phenazine-specific methyltransferase
PA4210*∫	*phzA1*		135.67	906.32	Probable phenazine biosynthesis protein
PA4211*∫	*phzB1*		481.60	2948.11	Probable phenazine biosynthesis protein
PA4212[#]	*phzC1*		24.42	160.42	Phenazine biosynthesis protein PhzC
PA4213[#]	*phzD1*		88.44	658.55	Phenazine biosynthesis protein PhzD
PA4214[#]	*phzE1*		55.10	383.29	Phenazine biosynthesis protein PhzE
PA4215[#]	*phzF1*		49.19	316.14	Probable phenazine biosynthesis protein
PA4216[#]	*phzG1*		21.28	135.57	Probable pyridoxamine 5'-phosphate oxidase
PA4217*∫	*phzS*		15.31	76.06	Flavin-containing monooxygenase
PA4219*	*ampO*			-3.50	AmpO

PA number[a]	Gene name[a]	PqsE[b]	RhlR[c]	PqsE+RhlR[d]	Product name[a]
PA4220*				-4.60	Hypothetical protein
PA4221*	*fptA*			-4.05	Fe(III)-pyochelin outer membrane receptor precursor
PA4222*				-3.67	Probable ATP-binding component of ABC transporter
PA4223*				-4.04	Probable ATP-binding component of ABC transporter
PA4224*	*pchG*			-4.33	Pyochelin biosynthetic protein PchG
PA4225*	*pchF*			-3.82	Pyochelin synthetase
PA4226*	*pchE*			-4.14	Dihydroaeruginoic acid synthetase
PA4227*	*pchR*			-2.59	Transcriptional regulator PchR
PA4228*	*pchD*			-6.48	Pyochelin biosynthesis protein PchD
PA4229*	*pchC*			-6.52	Pyochelin biosynthetic protein PchC
PA4230*	*pchB*			-6.18	Salicylate biosynthesis protein PchB
PA4231*	*pchA*			-5.56	Salicylate biosynthesis isochorismate synthase
PA4290				-2.43	Probable chemotaxis transducer
PA4296	*pprB*			2.16	Two-component response regulator, PprB
PA4306*	*flp*		2.19	6.28	Type IVb pilin, Flp
PA4384*∫			2.54	6.18	Hypothetical protein
PA4442	*cysN*			-2.11	ATP sulfurylase GTP-binding subunit/APS kinase
PA4443	*cysD*			-2.00	ATP sulfurylase small subunit
PA4467				-2.45	Hypothetical protein
PA4468	*sodM*			-2.64	Superoxide dismutase
PA4469				-2.40	Hypothetical protein
PA4470	*fumC1*			-2.57	fumarate hydratase
PA4590	*pra*			3.63	Protein activator
PA4591				2.33	Hypothetical protein
PA4594				2.03	Probable ATP-binding component of ABC transporter
PA4610*				-2.71	Hypothetical protein
PA4612*				2.48	Conserved Hypothetical protein
PA4613*∫	*katB*			3.51	Catalase
PA4675	*chtA*			-2.30	ChtA
PA4709*				-3.54	PhuS
PA4710	*phuR*			-3.80	Heme/Hemoglobin uptake outer membrane receptor PhuR precursor
PA4895				-2.09	Probable transmembrane sensor
PA4896				-2.66	Probable sigma-70 factor, ECF subfamily
PA4916*∫	*nrtR*		2.61	3.93	Nudix-related transcriptional regulator NrtR
PA4917∫	*nadD2*		3.08	4.39	Nicotinate mononucleotide adenylyltransferase NadD2
PA4918*	*pcnA*			-2.70	Hypothetical protein
PA5058*∫	*phaC2*			2.31	Poly(3-hydroxyalkanoic acid) synthase 2
PA5161∫	*rmlB*		2.92	4.65	dTDP-D-glucose 4,6-dehydratase
PA5162∫	*rmlD*			2.26	dTDP-4-dehydrorhamnose reductase
PA5163∫	*rmlA*		2.06	2.73	Glucose-1-phosphate thymidylyltransferase
PA5164∫	*rmlC*		2.02	2.67	dTDP-4-dehydrorhamnose 3,5-epimerase
PA5180				2.90	Conserved Hypothetical protein

PA number[a]	Gene name[a]	PqsE[b]	RhlR[c]	PqsE+RhlR[d]	Product name[a]
PA5181*			2.12	3.74	Probable oxidoreductase
PA5181.1				2.85	P34
PA5219ʃ			3.06	6.38	Hypothetical protein
PA5220*ʃ			8.49	20.53	Hypothetical protein
PA5221				2.88	Probable FAD-dependent monooxygenase
PA5352			2.00	4.22	Conserved Hypothetical protein
PA5353	*glcF*			3.55	Glycolate oxidase subunit GlcF
PA5354	*glcE*			3.31	Glycolate oxidase subunit GlcE
PA5355	*glcD*			3.36	Glycolate oxidase subunit GlcD
PA5356ʃ	*glcC*			2.38	Transcriptional regulator GlcC
PA5359				-2.33	Hypothetical protein
PA5368	*pstC*			-2.02	Membrane protein component of ABC phosphate transporter
PA5375	*betT1*			2.22	BetT1
PA5383			4.30	4.89	Conserved Hypothetical protein
PA5427*	*adhA*			-2.02	Alcohol dehydrogenase
PA5440				-2.48	Probable peptidase
PA5445				-2.31	Probable coenzyme A transferase
PA5460*				2.20	Hypothetical protein
PA5475*				-2.12	Hypothetical protein
PA5546				2.38	Conserved hypothetical protein

[a] PA number, gene name and product name are from the *Pseudomonas* Genome Database [102]. Class I genes, blue; Class II genes, red; Class III genes, black; genes not belonging to Classes I, II and III, green.

[b] Fold change in gene expression in the ΔQS-Eind(pHERD30T) strain grown in the LB supplemented with 0.1% (w/v) L-arabinose, 10 µM C_4-HSL and 500 µM IPTG (PqsE) compared with the same strain grown in the LB supplemented with 0.1% (w/v) L-arabinose and 10 µM C_4-HSL.

[c] Fold change in gene expression in the ΔQS-Eind(pHERD-*rhlR*) strain grown in the LB supplemented with 0.1% (w/v) L-arabinose and 10 µM C_4-HSL (RhlR) compared to the ΔQS-Eind(pHERD30T) strain grown in the LB supplemented with 0.1% (w/v) L-arabinose and 10 µM C_4-HSL.

[d] Fold change in gene expression in the ΔQS-Eind(pHERD-*rhlR*) strain grown in the LB supplemented with 0.1% (w/v) L-arabinose, 10 µM C_4-HSL and 500 µM IPTG (PqsE+RhlR) compared to the ΔQS-Eind(pHERD30T) strain grown in LB supplemented with 0.1% (w/v) L-arabinose and 10 µM C_4-HSL.

*, genes previously reported to be controlled by PqsE [12,13].

ʃ, genes previously reported to be controlled by RhlR [29,75].

[#], The *phz1* and *phz2* operons share 100% sequence identity from *phzC* to *phzG* [103], hence this analysis does not allow discriminating between the genes belonging to one or the other operon.

Table S2. Bacterial strains and plasmids used in this study

Strains/plasmids	Relevant characteristics and plasmids construction	Reference/Source
E. coli		
DH5α	Cloning strain	[104]
S17.1λ*pir*	Conjugative strain for suicide plasmids	[105]
P. aeruginosa		
PAO1	Nottingham collection wild type *P. aeruginosa* PAO1 strain	
PAO1*	Wild type strain of *P. aeruginosa* PAO1 ATCC 15692	
PAO1 Δ*pqsA PpqsA::lux* (AQ-Rep)	PAO1 derivative strain deleted in the *pqsA* gene carrying chromosomal insertion of the *PpqsA::lux* transcriptional fusion; used for the detection of 2-alkyl-4-quinolones (AQs) signal molecules.	[96]
PAO1 Δ*rhlI PrhlA::lux* (C_4-HSL-Rep)	PAO1* derivative strain deleted in the *rhlI* gene carrying the pMS402 plasmid containing the P*rhlA::lux* transcriptional fusion; used for the detection of *N*-butanoyl-homoserine lactone (C_4-HSL) signal molecule; Km^R.	[95]
PA14-R3	PA14 derivative strain deleted in the *lasI* gene carrying chromosomal insertion of the P*rsaL*::*lux* transcriptional fusion; used for the detection of *N*-(3-oxododecanoyl)-homoserine lactone ($3OC_{12}$-HSL) signal molecule.	[97]
Δ*rhlR*	PAO1 derivative strain carrying in frame clear deletion of the *rhlR* gene, obtained by allelic exchange using the plasmid pDM4Δ*rhlR.*	[14]
Δ4AQ	Quadruple mutant of PAO1 carrying in frame clear deletion of the *pqsA*, *pqsH* and *pqsL* genes, in which *pqsE* is under the control of an IPTG-inducible promoter.	[12]
Δ4AQΔ*rhl*	Δ4AQ derivative strain carrying in frame clear deletion of the *rhlI-rhlR* gene *locus*, obtained by allelic exchange using the plasmid pDM4Δ*rhl*.	This study
Δ4AQΔ*rhl*Δ*las*	Δ4AQΔ*rhl* derivative strain carrying in frame clear deletion of the *lasR-rsaL-lasI* gene *locus*, obtained by allelic exchange using the plasmid pDM4Δ*las*.	This study
ΔQS-Eind	Δ4AQΔ*rhl*Δ*las* derivative strain carrying in frame clear deletion of the *phnA-phnB-pqsR* gene *locus,* obtained by allelic exchange using the plasmid pDM4Δ*phnAB-pqsR*.	This study
Δ5	PAO1* derivative strain carrying in frame clear deletion of the *lasR*, *rsaL*, *lasI*, *rhlI*, *rhlR*, and *pqsE* genes, obtained by stepwise mutagenesis using the plasmids pDM4Δ*las*, pDM4Δ*rhl*, and pDM4Δ*pqsE.*	This study
ΔQS	PAO1* derivative strain carrying in frame clear deletions of the *lasR*, *rsaL*, *lasI*, *rhlI*, *rhlR, pqsA*, *pqsB*, *pqsC*, *pqsD*, *pqsE*, *phnA*, *phnB*, *pqsR*, *pqsH*, *pqsL* genes, obtained by stepwise mutagenesis using the plasmids pDM4Δ*las*, pDM4Δ*rhl*, pDM4Δ*pqsABCDE-phnAB-pqsR*, pDM4Δ*pqsH*, and pDM4Δ*pqsL.*	This study
Δ*rhlI*	PAO1* derivative strain carrying in frame clear deletion of the *rhlI* gene, obtained by allelic exchange using the plasmid pDM4Δ*rhlI*.	This study
Δ*pqsE*	PAO1* derivative strain carrying in frame clear deletion of the *pqsE* gene, obtained by allelic exchange using the plasmid pDM4Δ*pqsE*.	This study
Δ*rhlI*Δ*pqsE*	PAO1* derivative strain carrying in frame clear deletions of the *rhlI* and *pqsE* genes, obtained by stepwise mutagenesis using the plasmids pDM4Δ*rhlI* and pDM4Δ*pqsE*.	This study
**Plasmids**		
pDM4	Suicide vector; *sacBR*; *oriR6K*; Cm^R.	[93]
pDM4Δ*pqsE*	pDM4 derivative plasmid for *pqsE* in-frame deletion; Cm^R.	[14]
pDM4Δ*pqsH*	pDM4 derivative plasmid for *pqsH* in-frame deletion; Cm^R.	[96]
pDM4Δ*pqsL*	pDM4 derivative plasmid for *pqsL* in-frame deletion; Cm^R.	[12]
pUCP18	pUC18 derivative containing a stabilizing fragment for maintenance in *Pseudomonas*; Ap^R, *E. coli* / Cb^R, *P. aeruginosa*.	[106]
pUCP-*pqsE*	pUCP18 derivative for *pqsE* complementation; Ap^R.	[14]
pHERD30T	Plasmid for L-arabinose-inducible protein expression in *P. aeruginosa*; Gm^R.	[44]
miniCTX-*lux*	Promoter-probe vector containing the *luxCDABE* operon; Tc^R.	[100]
miniCTX-P*pqsA::lux*	miniCTX-lux derivative used to insert the P*pqsA::lux* fusion in the chromosome of different *P. aeruginosa* strains; Tc^R.	[101]
pDM4Δ*pqsABCDE-phnAB-pqsR*	pDM4-derived plasmid for the generation of the ΔQS mutant strain; Cm^R. It contains the DNA fragments encompassing the upstream region of *pqsA* gene originated with primers FW*pqsA*UP and RV*pqsA*UP (Table S3) and the upstream region of the *pqsR* gene originated with primers FW*pqsR*UP and RV*pqsR*UP (Table S3), and cloned in pDM4 by XhoI-XbaI.	This study

Strains/plasmids	Relevant characteristics and plasmids construction	Reference/Source
pDM4Δ*phnAB-pqsR*	pDM4-derived plasmid for the generation of the ΔQS-Eind mutant strain; Cm^R. It contains the DNA fragments encompassing the upstream region of the *phnA* gene originated with primers FW*phnA*UP and RV*phnA*UP (Table S3) and the upstream region of the *pqsR* gene originated with primers FW*pqsR*UP and RV*pqsR*UP (Table S3), and cloned in pDM4 by XhoI-XbaI.	This study
pDM4Δ*rhlI*	pDM4-derived plasmid for the generation of the Δ*rhlI* and Δ*rhlI*Δ*pqsE* mutant strains; Cm^R. It contains the DNA fragments encompassing the upstream region of the *rhlI* gene originated with primers FW*rhlI*UP and RV*rhlI*UP (Table S3) and the downstream region of the *rhlI* gene originated with primers FW*rhlI*DOWN and RV*rhlI*DOWN2 (Table S3), and cloned in pDM4 by XbaI-XhoI.	This study
pDM4Δ*rhl*	pDM4-derived plasmid for the generation of the Δ*las*Δ*rhl* and ΔQS-Eind mutant strains; Cm^R. It contains the DNA fragments encompassing the upstream region of the *rhlR* gene originated with primers FW*rhlR*UP and RV*rhlR*UP (Table S3) and the downstream region of the *rhlI* gene originated with primers FW*rhlI*DOWN and RV*rhlI*DOWN (Table S3), and cloned in pDM4 by XbaI-ApaI.	This study
pDM4Δ*las*	pDM4-derived plasmid for the generation of the Δ*las*Δ*rhl* and ΔQS-Eind mutant strains; Cm^R. It contains the DNA fragments encompassing the upstream region of the *lasR* gene originated with primers FW*lasR*UP and RV*lasR*UP (Table S3) and the downstream region of the *lasI* gene originated with primers FW*lasI*DOWN and RV*lasI*DOWN (Table S3), and cloned in pDM4 by ApaI-XhoI.	This study
pUCP-*rhlR*	pUCP18 derivative for *rhlR* complementation; Ap^R. Obtained by cloning in pUCP18 with restriction enzymes EcoRI-HindIII a DNA region encompassing the *rhlR* gene amplified with primers FW*rhlR* and RV*rhlR* (Table S3).	This study
pUCP-*rhlI*	pUCP18 derivative for *rhlI* complementation; Ap^R. Obtained by cloning in pUCP18 with restriction enzymes SacI-SmaI a DNA region encompassing the *rhlR* gene amplified with primers FW*rhlI* and RV*rhlI* (Table S3).	This study
pHERD-*rhlR*	pHERD30T derivative for the L-arabinose-dependent expression of *rhlR;* Gm^R. Obtained by cloning in pHERD30T with restriction enzymes EcoRI-HindIII a DNA region encompassing the *rhlR* gene amplified with primers FW*rhlR* and RV*rhlR* (Table S3).	This study
miniCTX-P*rhlA::lux*	miniCTX-lux derivative used to insert the P*rhlA::lux* fusion in the chromosome of different *P. aeruginosa* strains; Tc^R. Obtained by cloning in miniCTX-*lux* with restriction enzymes XhoI-BamHI a DNA region encompassing the P*rhlA* promoter amplified with primers FWP*rhlA* and RVP*rhlA* (Table S3).	This study
miniCTX-P*phzM::lux*	miniCTX-lux derivative used to insert the P*phzM::lux* fusion in the chromosome of different *P. aeruginosa* strains; Tc^R. Obtained by cloning in miniCTX-*lux* with restriction enzymes EcoRI-BamHI a DNA region encompassing the P*rhlA* promoter amplified with primers FWP*phzM* and RVP*phzM* (Table S3).	This study
miniCTX-P*mexG::lux*	miniCTX-lux derivative used to insert the P*mexG::lux* fusion in the chromosome of different *P. aeruginosa* strains; Tc^R. Obtained by cloning in miniCTX-*lux* with restriction enzymes XhoI-SmaI a DNA region encompassing the P*mexG* promoter amplified with primers FWP*mexG* and RVP*mexG* (Table S3).	This study
miniCTX-P_{PA2274}*::lux*	miniCTX-lux derivative used to insert the P_{PA2274}*::lux* fusion in the chromosome of different *P. aeruginosa* strains; Tc^R. Obtained by cloning in miniCTX-*lux* with restriction enzymes HindIII-BamHI a DNA region encompassing the P_{PA2274} promoter amplified with primers FWP_{PA2274} and RVP_{PA2274} (Table S3).	This study
miniCTX-P*vqsR::lux*	miniCTX-lux derivative used to insert the P*vqsR::lux* fusion in the chromosome of different *P. aeruginosa* strains; Tc^R. Obtained by cloning in miniCTX-*lux* with restriction enzymes EcoRI-BamHI a DNA region encompassing the P*vqsR* promoter amplified with primers FWP*vqsR* and RVP*vqsR* (Table S3).	This study
miniCTX-P*hsiA2::lux*	miniCTX-lux derivative used to insert the P*hsiA::lux* fusion in the chromosome of different *P. aeruginosa* strains; Tc^R. Obtained by cloning in miniCTX-*lux* with restriction enzymes XhoI-SmaI a DNA region encompassing the P*hsiA2* promoter amplified with primers FWP*hsiA2* and RVP*hsiA2* (Table S3).	This study
miniCTX-P*clpP2::lux*	miniCTX-lux derivative used to insert the P*clpP2::lux* fusion in the chromosome of different *P. aeruginosa* strains; Tc^R. Obtained by cloning in miniCTX-*lux* with restriction enzymes XhoI-SmaI a DNA region encompassing the P*clpP2* promoter amplified with primers FWP*clpP2* and RVP*clpP2* (Table S3).	This study
miniCTX-P_{PA1131}*::lux*	miniCTX-lux derivative used to insert the P_{PA1131}*::lux* fusion in the chromosome of different *P. aeruginosa* strains; Tc^R. Obtained by cloning in miniCTX-*lux* with restriction enzymes XhoI-SmaI a DNA region encompassing the P_{PA1131} promoter amplified with primers FWP_{PA1131} and RVP_{PA1131} (Table S3).	This study

Strains/plasmids	Relevant characteristics and plasmids construction	Reference/Source
miniCTX-P*pvdS::lux*	miniCTX-lux derivative used to insert the P*pvdS::lux* fusion in the chromosome of different *P. aeruginosa* strains; Tc^R. Obtained by cloning in miniCTX-*lux* with restriction enzymes EcoRI-BamHI a DNA region encompassing the P*pvdS* promoter amplified with primers FWP*pvdS* and RVP*pvdS* (Table S3).	This study
miniCTX-P*pchR::lux*	miniCTX-*lux* derivative used to insert the P*pchR::lux* fusion in the chromosome of different *P. aeruginosa* strains; Tc^R. Obtained by cloning in miniCTX-*lux* with restriction enzymes EcoRI-BamHI a DNA region encompassing the P*pchR* promoter amplified with primers FWP*pchR* and RVP*pchR* (Table S3).	This study

Additional references not included in the main text:

104. Grant SG, Jessee J, Bloom FR, Hanahan D. 1990. Differential plasmid rescue from transgenic mouse DNAs into *Escherichia coli* methylation-restriction mutants. Proc Natl Acad Sci U S A 87:4645-4649.

105. Simon R, Priefer U, Puhler A. 1983. A broad host range mobilization system for *in vivo* genetic-engineering: transposon mutagenesis in Gram-negative bacteria. Biotechnology 1:784-791.

106. Schweizer HP. 1991. *Escherichia-Pseudomonas* shuttle vectors derived from pUC18/19. Gene 97:109-121.

Table S3. Oligonucleotides used in this study

Name	Sequence (5'-3')[a]	Restriction site
FW*pqsA*UP	ACGCGTCGACCCAGTGTACTACGCAATGG	SalI
RV*pqsA*UP	CCGGAATTCTGTGGACATGACAGAACGTTC	EcoRI
FW*phnA*UP	CCGCTCGAGGTTTTCTACGACGTGCGAC	XhoI
RV*phnA*UP	CCGGAATTCCGCGCCCATGGGCGACT	EcoRI
FW*pqsR*UP	TGCTCTAGAACAAAAGACATAGGTTTCGGT	XbaI
RV*pqsR*UP	CCGGAATTCAATAGGCATCCCTTATTCCTTT	EcoRI
FW*rhlR*UP	TGCTCTAGAAGGAACCGCTGCGCGGC	XbaI
RV*rhlR*UP	CCCAAGCTTCCTCATTGCAGTAAGCCC	HindIII
FW*rhlI*UP	ATATCTAGAAGCGTGCTTTCCGTGGCG	XbaI
RV*rhlI*UP	ATAAAGCTTTTCGATCATGACCAAGTCCC	HindIII
FW*rhlI*DOWN	ATAAAGCTTGCGGTGTGAGGTCGTCAG	HindIII
RV*rhlI*DOWN	ACCGGGCCCGGCGTCTTGCCGTCGC	ApaI
RV*rhlI*DOWN2	ATACTCGAGGTAGGGAATCGAGAAATACG	XhoI
FWlas*R*UP	ATAGGGCCCGCCGAACTGGAAAAGTGGC	ApaI
RV*lasR*UP	ATAAAGCTTCAAGGCCATAGCGCTACGT	HindIII
FW*lasI*DOWN	ATAAAGCTTGTTTCATGACGGGGACCTG	HindIII
RV*lasI*DOWN	ATACTCGAGGCGCATCCGCAGGAGGCG	XhoI
FW*rhlR*	TATGAATTCATGAGGAATGACGGAGGCTT	EcoRI
RV*rhlR*	TATAAGCTTTCAGATGAGACCCAGCGCC	HindIII
FW*rhlI*	CGAGCTCATGATCGAATTGCTCTCTGAA	SacI
RV*rhlI*	TCCCCCGGGTCACACCGCCATCGACAG	SmaI
FW*pqsB*	CCGCTCGAGCGACCAGGGCTATCGCA	XhoI
RV*pqsB*	CCGGAATTCCTTATGCATGAGCTTCTCC	EcoRI
FW16SRT	GAGAGTTTGATCCTGGCTCAG	
RV16SRT	CTACGGCTACCTTGTTACGA	
FW*rhlR*RT	TGGGCTTCGATTACTACGCC	
RV*rhlR*RT	TCGCTCCAGACCACCATTTC	
FW*pqsE*RT	CGGTGTTCCTGCTGCGTC	
RV*pqsE*RT	GACGCCAGGACCTGTACG	
FWPA2827RT	AGTTCCACATCTGTCGCCTG	
RVPA2827RT	GTGGCTGAAGTCGTCCAGTT	
FWPA2828RT	CCTGTCCAAATCCTACCGGG	
RVPA2828RT	GGCAGGACCAGATCGTTGAT	
FW*bexR*RT	GGAGATCGTCCTGCAGATGG	
RV*bexR*RT	CAGTTGTTCGAGGGTGTCCA	
FWPA1203RT	CTTATCGCGTGACCCTCGG	
RVPA1203RT	AGTTGCACGTCGATACCCTC	
FW*qteE*RT	GATGCGGTGAGCGACTACAT	
RV*qteE*RT	GAAGATGCTGGTTGGCATCG	
FW*clpP2*RT	GATCCGCTTCATCACACCGA	
RV*clpP2*RT	TCCTTCATCCGCACGATCTC	
FW*vqsR*RT	AACCCTATGCCTGTCGTTGG	
RV*vqsR*RT	CGGCGATATCAACCCTCTCC	
FW*hsiB2*RT	TCGGCATCGACAAGATGACC	
RV*hsiB2*RT	ATCAGCTTCTTCAGCTCGGG	
FW*mpaR*RT	CCGGTGGAAGAACTGGTCAT	
RV*mpaR*RT	CACTTCCAGGTCGATGCCTT	

Name	Sequence (5'-3')[a]	Restriction site
FWPA3329RT	GCCAGGGCCATCATTTCAAC	
RVPA3329RT	GAGATGCCGGTGAGGAACTG	
FW*nosR*RT	GCTATCGGTGGTCAACGTG	
RV*nosR*RT	GCGAGTTCGTTGAGCAGTTC	
FW*chiC*RT	GCACAAGAACGGCAAGTACG	
RV*chiC*RT	CAGGCTCTCGGTGAGGTAGT	
FW*lecA*RT	CAGGGCAGGTAACGTCGATT	
RV*lecA*RT	CAACCCGGTATTGACCGGAA	
FW*mexG*RT	CTGGCGAAGCTGTTCGACTA	
RV*mexG*RT	TTGCTCCAGAAGGTGTGGAC	
FW*rhlA*RT	CATCTGCTCAACGAGACCGT	
RV*rhlA*RT	TGCCGTTGATGAAATGCACG	
FW*phzM*RT	ACATGGTGCTGTTCTACGGC	
RV*phzM*RT	CCTGGGGATCTCGTGGAATG	
FWPA2069RT	TACTTCTACGGGCGCATCAC	
RVPA2069RT	GATCGCTGTAGCCGTCGTAG	
FW*phzD*RT	CGTGCAGATCGCCTACACC	
RV*phzD*RT	CAGGTCGGAGTGGAAGAAGG	
FW*pvdS*RT	GGAACAACTGTCTACCCGCA	
RV*pvdS*RT	GTAGCTGAGCTGTGCCTTGA	
FW*pvdA*RT	AGTGGCAAGCCGATGAAGAT	
RV*pvdA*RT	GTTCGCGGCTGTAGATGAGA	
FW*pvdQ*RT	GAAGACGCTCGAGGAGATGG	
RV*pvdQ*RT	TGAAGCGCTGGAAGTAGACG	
FW*pchA*RT	CTACCGGGTACTCTGCCAAC	
RV*pchA*RT	GCTCACCTTGGCTTCCCATT	
FW*pchR*RT	CTCAGCGCACAGTTCCTTTC	
RV*pchR*RT	CGAACACCTTGCGAAAGCC	
FW*catA*RT	GCCAATACCCAAGGCACCTA	
RV*catA*RT	GAAGAAGTGAATGTGCGCCG	
FW*metE*RT	CTACCATCGGCTCTTTCCCG	
RV*metE*RT	GGCGAAGTACTCGACCATGT	
FW*phzH*RT	TTCGTTCTCGGTGGACTTCG	
RV*phzH*RT	GTCGCCGAAGGTGAAAGGTA	
FWP*rhlA*	CCGCTCGAGAGGCCTGCGAAGTGTCCT	XhoI
RVP*rhlA*	CGCGGATCCCCGCATTTCACACCTCCCAA	BamHI
FWP*phzM*	CCGGAATTCCGCCGCCTCCGAGAGGG	EcoRI
RVP*phzM*	CGCGGATCCATTCATCTTTTATTCTCTCTCGTT	BamHI
FWP*mexG*	CCGCTCGAGACGGCAAGTCCTGTAGGGC	XhoI
RVP*mexG*	TCCCCCGGGCTGCATGGGTCGTTCCTTGT	SmaI
FWPPA2274	CCCAAGCTTTCGGCGGAAAGCTGGTCGC	HindIII
RVPPA2274	CGCGGATCCGAGCATGGCGGGTTACTCC	BamHI
FWP*vqsR*	CCGGAATTCCGATGAAGTCATACCAGTTGTA	EcoRI
RVP*vqsR*	CGCGGATCCCAATGCGATATCCACACAACTA	BamHI
FWP*hsiA2*	CCGCTCGAGGGCCAGTATTTTCAAGGGCT	XhoI
RVP*hsiA2*	TCCCCCGGGATAGGTCATCCTAACCCTTCA	SmaI
FWP*clpP2*	CCGCTCGAGACGAACCATCCCTGCGTCC	XhoI
RVP*clpP2*	TCCCCCGGGGGTTTTCATGCAACCTCCTG	SmaI
FWPPA1131	CCGCTCGAGCTGAACAGCTCGGAGTAC	XhoI

Name	Sequence (5’-3’)[a]	Restriction site
RVPPA1131	TCCCCCGGGCGTGGACACGCCAGGATC	SmaI
FWP*pvdS*	CCGGAATTCTCACAGAGGGGAACGGATAA	EcoRI
RVPpvdS	CGCGGATCCTTCCGACATGGAAATCACCTT	BamHI
FWP*pchR*	CCGGAATTCGTCGTCGCAGAGCGCGAT	EcoRI
RVP*pchR*	CGCGGATCCGGTCATCAGGTTTTCCTGTAG	BamHI

[a] Restriction sites are underlined in the primer sequences.
